# *Drosophila* Interspecific Hybrids Phenocopy piRNA-Pathway Mutants

**DOI:** 10.1371/journal.pbio.1001428

**Published:** 2012-11-20

**Authors:** Erin S. Kelleher, Nathaniel B. Edelman, Daniel A. Barbash

**Affiliations:** Department of Molecular Biology and Genetics, Cornell University, Ithaca, New York, United States of America; Duke University, United States of America

## Abstract

The Piwi-interacting RNA (piRNA) pathway defends the germline of animals from the deleterious activity of selfish transposable elements (TEs) through small-RNA mediated silencing. Adaptation to novel invasive TEs is proposed to occur by incorporating their sequences into the piRNA pool that females produce and deposit into their eggs, which then propagates immunity against specific TEs to future generations. In support of this model, the F1 offspring of crosses between strains of the same *Drosophila* species sometimes suffer from germline derepression of paternally inherited TE families, caused by a failure of the maternal strain to produce the piRNAs necessary for their regulation. However, many protein components of the *Drosophila* piRNA pathway exhibit signatures of positive selection, suggesting that they also contribute to the evolution of host genome defense. Here we investigate piRNA pathway function and TE regulation in the F1 hybrids of interspecific crosses between *D. melanogaster* and *D. simulans* and compare them with intraspecific control crosses of *D. melanogaster*. We confirm previous reports showing that intraspecific crosses are characterized by derepression of paternally inherited TE families that are rare or absent from the maternal genome and piRNA pool, consistent with the role of maternally deposited piRNAs in shaping TE silencing. In contrast to the intraspecific cross, we discover that interspecific hybrids are characterized by widespread derepression of both maternally and paternally inherited TE families. Furthermore, the pattern of derepression of TE families in interspecific hybrids cannot be attributed to their paucity or absence from the piRNA pool of the maternal species. Rather, we demonstrate that interspecific hybrids closely resemble piRNA effector-protein mutants in both TE misregulation and aberrant piRNA production. We suggest that TE derepression in interspecific hybrids largely reflects adaptive divergence of piRNA pathway genes rather than species-specific differences in TE-derived piRNAs.

## Abstract 2

Hybrids of two *Drosophila* species show transposable element derepression and piRNA pathway malfunction, revealing adaptive evolution of piRNA pathway components.

## Author Summary

Eukaryotic genomes contain large quantities of transposable elements (TEs), short self-replicating DNA sequences that can move within the genome. The selfish replication of TEs has potentially drastic consequences for the host, such as disruption of gene function, induction of sterility, and initiation or exacerbation of some cancers. Like the adaptive immune system that defends our bodies against pathogens, the Piwi-interacting RNA (piRNA) pathway defends animal genomes against the harmful effects of TEs. Fundamental to piRNA-mediated defense is the production of small noncoding RNAs that act like antibodies to target replicating TEs for destruction by piRNA-effector proteins. piRNAs are expected to diverge rapidly between species in response to genome infection by increasingly disparate TEs. Here, we tested this hypothesis by examining how differences in piRNAs between two species of fruit fly relate to TE “immunity” in their hybrid offspring. Because piRNAs are maternally deposited, we expected excessive replication of paternal TEs in hybrids. Surprisingly, we observe increased activity of both maternal and paternal TEs, together with defects in piRNA production that are reminiscent of piRNA effector-protein mutants. Our observations reveal that piRNA effector-proteins do not function properly in hybrids, and we propose that adaptive evolution among piRNA effector-proteins contributes to host genome defense and leads to the functional incompatibilities that we observe in hybrids.

## Introduction

Transposable elements (TEs) are ubiquitous, mobile genetic parasites that often are exceptionally deleterious to their hosts. TE insertions can mediate ectopic recombination that results in chromosomal rearrangements, and can also disrupt functional DNA sequences (reviewed in [1],[2]). Unrestricted TE propagation, furthermore, is associated with severe pathologies including tumorigenesis, tumor progression, gonadal atrophy, and sterility [3]–[6]. Despite these costs, many TE families are extraordinarily successful parasites: they achieve high copy numbers within host genomes [7],[8] and can invade novel hosts through horizontal transfer [9]–[12]. Host genomes are therefore continually challenged to suppress a potent and dynamic suite of TEs.

The selfish replication of TEs in the germline is of central importance for both the parasite and its host, as novel insertions and associated mutations in gametes are heritable. Recent studies have revealed that in metazoan germlines, the Piwi-interacing RNA pathway (piRNA pathway) acts as a genomic immune system, mediating transcriptional and post-transcriptional silencing of endogenous and invasive TEs (reviewed in [13],[14]). Though less well characterized, piRNA-mediated silencing also plays a role in the regulation of some protein-coding genes [15],[16]. The mechanism of piRNA-mediated silencing is best understood in *Drosophila,* where it is defined by short, antisense, TE-derived RNAs (piRNAs, 23–29 nt) that are found in complexes with three Piwi-clade Argonaute proteins: Piwi, Aubergine (Aub), and Argonaute-3 (Ago3) [17].

The *Drosophila* genome is postulated to acquire immunity to an invasive TE family by incorporating its sequence into piRNA clusters [18]–[20]. These TE-rich genomic regions are transcribed into long precursor transcripts and then processed into mature, 23–29 nt piRNAs [17],[21]. For many TE families, maternal deposition of piRNAs is thought to be critical for propagating piRNA-mediated TE silencing in the offspring germline [18]. Indeed, the failure of the maternal cytotype to deposit piRNAs derived from specific TE families is a cause of hybrid dysgenesis, a syndrome of germline TE derepression in crosses between strains of the same *Drosophila* species [22]–[25]. Hybrid dysgenesis occurs when the paternal but not maternal strain carries active members of a TE family [26]–[28]. piRNAs derived from the dysgenic TE family are rare or absent in the maternal cytotype because active TE copies are found exclusively in the paternal genome. Multigenerational dysgenic crosses between *D. melanogaster* strains indicate that maternally mediated silencing is rapidly achieved within ∼6 generations as piRNA cluster insertions are transmitted through females [29].

The piRNA-dependent model of host genome adaptation is appealing because it allows for the acquisition of silencing through TE insertions into piRNA clusters, without necessitating changes in protein coding genes. Intriguingly, however, many *Drosophila* piRNA-pathway effector proteins exhibit signatures of adaptive evolution [30]–[33] suggesting that protein divergence also plays a role in the evolution of host genome defense. F1 interspecific hybrids provide a unique opportunity to test this hypothesis, as piRNA pathway function in these animals is potentially impacted by interspecific divergence in both the piRNA pool and in piRNA-effector proteins. If TE-driven adaptation is confined solely to the piRNA pool, patterns of TE derepression in interspecific hybrids should be qualitatively similar compared to intraspecific hybrids but quantitatively more extreme. Therefore, two specific predictions can be made about TE derepression in interspecific hybrids: (1) Because maternal deposition is critical for silencing, derepressed TE families should be rare among the ovarian piRNAs of the maternal species when compared to the paternal species, and (2) TE families that are active solely in the paternal but not maternal species should be exceptionally prone to derepression.

Here we test these hypotheses by comparing TE silencing and piRNA production between the F1 female offspring from intraspecific and interspecific crosses of *Drosophila*. The intraspecific hybrids we examined were from crosses between two strains of *D. melanogaster*, one of which contains an active *I-element* that induces intraspecific hybrid dysgenesis. We compare these results to interspecific hybrids from crosses between *D. melanogaster* and its sibling species *D. simulans* (MRCA ∼3–5 MYA, [34]). A range of well-characterized TE classes, including both common and species-specific elements [10], makes these species a powerful model for investigating how germline TE silencing diverges in response to dynamic changes in genomic TE content. Furthermore, the >6 million years of divergence between these two species have allowed considerable functional differences to accrue among protein coding genes (for example, [35]–[37]).

## Results

### Global TE Derepression in *Hmr-*Rescued Interspecific Hybrids

Hybrid females of *D. melanogaster* and *D. simulans* are normally sterile and agametic, which confounds examination of germline TE activity and piRNA pathway function. We therefore examined hybrid females heterozygous for the dominant *Hmr* mutation, which partially rescues female fertility but does not appear to affect piRNA pathway function in the heterozygous state (Figure S1) [38]. We employed Illumina deep sequencing to compare mRNA transcript abundance in ovaries from interspecific hybrids to their parental pure species. We found very few misregulated protein-coding genes: Among 8,442 protein-coding genes represented in our mRNA-seq data, only 21 are underexpressed and 38 overexpressed in interspecific hybrids at the level of ≥2-fold differences in transcript abundance when compared to both *D. melanogaster* (*Hmr*/+) and *D. simulans* (*w^501^*) pure species (Table S1). Only one of these genes has a known role in piRNA regulation, *Brother of Yb* (*BoYb*), a recently characterized component of the germline piRNA pathway [39]. *BoYb* transcript abundance in interspecific hybrids was ∼25% and ∼36% compared to *D. melanogaster* and *D. simulans*, respectively, demonstrating reduced but not silenced expression (Table S2). These results strongly suggest that hybrid ovaries are similar to wild-type ovaries in their overall physiology and development.

**Figure 1:**
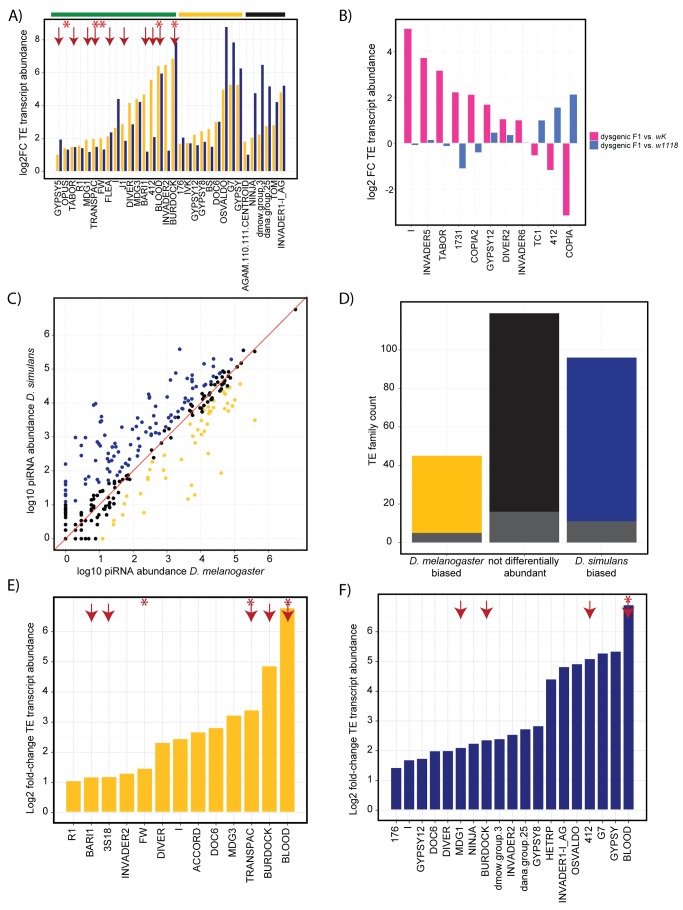
The pattern of TE derepression in interspecific hybrid ovaries does not correlate with interspecific differences in piRNA abundance and does not match expectations from models of intraspecific hybrid dysgenesis. (A) Widespread misexpression of TEs in interspecific hybrids. Log2 transformed ratio of TE transcripts exhibiting ≥2-fold increased transcript abundance in interspecific F1 hybrids relative to both *D. melanogaster* (yellow) and *D. simulans* (blue). Candidate horizontally transferred TE families that are derepressed in interspecific hybrids are highlighted by a red arrow [10] and/or a red asterisk [12]. TE families represented by full-length copies in both genomes, the *D. melanogaster* genome only, or neither genome are indicated under the green, yellow, and black bars, respectively [10],[49]. (B) No TEs in dysgenic intraspecific hybrids exhibit ≥2-fold increased transcript abundance relative to both parental strains. Log2 transformed fold-change in TE transcript abundance in F1 dysgenic hybrids relative to *D. melanogaster w^K^* (pink) and *w^1118^* (blue) parental strains. (C) Many TE classes have differential abundance of their corresponding piRNAs between *D. melanogaster* and *D. simulans*. *D. melanogaster* biased TEs (≥2-fold higher abundance) are yellow, and *D. simulans* biased TEs are shaded blue. Red line indicates equivalent expression values in both species. (D) TE derepression in interspecific hybrid ovaries does not correlate with interspecific differences in piRNA abundance. Each bar represents the total number of TE classes with the pattern of interspecific differential abundance for piRNAs from (C), while grey shading indicates the proportion that are misregulated in interspecific hybrids from (A). The proportion of misregulated TEs is not significantly different among the three classes (*X^2^* = 2.08, *df* = 2, *p* = 0.35). (E and F) Species-specific TE transcripts are equivalently derepressed from the maternal and paternal genomes in interspecific hybrids. Log2 transformed ratio of TE transcripts exhibiting ≥2-fold increased abundance in hybrids relative to *D. melanogaster* (yellow) or *D. simulans* (blue) when considering reads that map exclusively to the *D. melanogaster* or *D. simulans* genomes. Red arrows and asterisks as in (A).

We identified 32 out of 265 TE families that exhibit a >2-fold increase in transcript abundance in the ovaries of F1 interspecific hybrids (*Hmr/*+) relative to both parental pure species (Figure 1A). This represents a significantly higher incidence of overexpression than we observe among protein-coding genes, where only 38 of 8,442 examined genes are derepressed (*G-test* of independence = 132.96, *p* = 9.23e-31). While the degree of correlation between TE transcript abundance and transposition rate remains unknown, transcript abundance is a direct indicator of the efficacy of transcriptional and post-transcriptional silencing and increases in all piRNA-effector-protein mutant backgrounds [40]–[48]. Therefore, it provides the most relevant indicator of the robustness of piRNA-mediated TE regulation in interspecific hybrids.

Interestingly, transcriptional derepression in interspecific hybrids includes TE families regulated by either the germline or somatic piRNA pathways (Table S3). Derepression of somatically regulated elements is unexpected, because maternally deposited piRNAs are not posited to play a role in silencing these TE families [18]. Furthermore, we found that all derepressed TE families are represented by at least partial copies in both the *D. melanogaster* and *D. simulans* genomes [49], and none are among the five TE families previously identified as being potentially active only in *D. simulans* (Table S3) [10]. Thus, TE derepression in interspecific hybrids is not consistent with interspecific differences in active TE families.

### Patterns of TE Derepression Are Distinct in Interspecific Versus Intraspecific Hybrids

As an intraspecific comparison, we also examined TE transcript abundance in the F1 offspring of crosses between strains of *D. melanogaster* that differ in the presence of active *I-elements*. The *I-element* is a non-long-terminal repeat (non-LTR) retrotransposon that is represented by 10–15 active copies and a few inactive copies in the *D. melanogaster w^1118^* strain, while only inactive copies are found in the *D. melanogaster w^K^* strain [27]. When *w^K^* females are crossed to *w^1118^* males, the F1 female offspring exhibit sterility and germline derepression of the *I-element* [23]. *I-element* dysgenic ovaries present an ideal intraspecific control because their wild-type morphology allows for robust examination of piRNA pathway function. In contrast, *D. melanogaster P-element* dysgenesis is characterized by an early disruption of oogenesis that results in complete gonadal atrophy and a dramatic reduction in the number of germline cells [50]. We further consider similarities and differences among different intraspecific hybrid dysgenesis systems in Text S1.

Multiple TE families exhibit ≥2-fold transcript abundance in F1 dysgenic ovaries when compared to either *w^K^* (eight TE families) or *w^1118^* (three TE families). However, no TE families exhibit significantly higher abundance (≥2-fold) in dysgenic ovaries compared to both parental strains, including the *I-element* itself (Figure 1B). The lack of dramatic transcriptional derepression in the *I-element* is consistent with a previous report [51]. Global TE derepression, therefore, is a unique feature of interspecific hybrids.

### TE Derepression in Interspecific Hybrids Is Not Explained by Differences in the piRNA Pool

In intraspecific hybrid dysgenesis, TE mobilization in the F1 germline is associated with a failure of the maternal cytotype to deposit sufficient piRNAs to propagate piRNA silencing of a paternally inherited TE family. For example, *I-element*-derived piRNAs are 21-fold less abundant in ovaries from the *w^K^* strain compared to *w^1118^*, explaining why dysgenesis occurs when *w^K^* is the maternal parent [23]. To test the hypothesis that TE derepression in interspecific hybrids is caused by reduced abundance of their corresponding piRNAs in the *D. melanogaster* maternal cytotype, we employed deep-sequencing of ovarian small RNAs (18–30 nt) and identified 102 TE families that exhibit ≥2-fold reduced abundance in *D. melanogaster* piRNAs (≥23 nt) relative to *D. simulans* piRNAs (Figure 1C). Contrary to our expectation, TE families that are less abundant among *D. melanogaster* ovarian piRNAs are not more likely to be derepressed in interspecific hybrids than elements that exhibit no interspecific difference in abundance between the piRNA pools (Figure 1D, *z* value = −0.44, *df* = 257, *p* = 0.66). The reciprocal is also true: TE classes that are more abundant among *D. melanogaster* piRNAs, the maternal genotype, are not less likely to be derepressed in interspecific hybrids than non-differentially abundant elements (*z*-value = −0.55, *df* = 155, *p* = 0.58).

**Figure 2:**
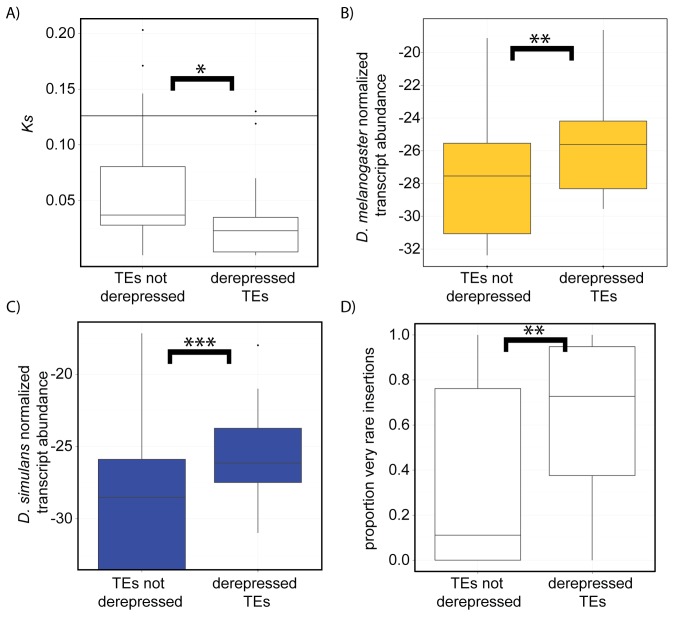
TE families derepressed in interspecific hybrids exhibit low silent divergence, high ovarian expression levels, and high transposition rates. (A) Silent site sequence divergence (*Ks*) between *D. melanogaster* and *D. simulans* of derepressed TEs is lower than non-derepressed TEs in interspecific hybrids. (B and C) Log2 TE-derived transcript abundance in *D. melanogaster* (B) and *D. simulans* (C) in ovarian mRNAs is compared for TE families not derepressed and derepressed in interspecific hybrids. TE transcript abundance was normalized by library size and the length of the consensus sequence for each TE family. (D) The proportion of TE insertions segregating at very low frequency (∼<1.5%) in North American *D. melanogaster* populations [52],[53] is higher for TE classes that are derepressed in interspecific hybrids. For all panels, TEs derepressed are as defined in Figure 1A. * Wilcoxon Rank Sum *p*<0.05. ** Wilcoxon Rank Sum *p*<0.01. *** Wilcoxon Rank Sum *p*<0.001.

One potential explanation for the absence of a relationship between interspecific differences in TE-derived piRNA abundance and derepression of those TEs in interspecific hybrids is that, within a TE family, sequence divergence between maternal piRNAs and paternal transcripts decreases the efficacy of silencing. Surprisingly, when we examined TE-derived reads that are uniquely assignable to either the *D. melanogaster* or the *D. simulans* genome (zero mismatches), both maternal and paternal transcripts exhibit increased abundance in interspecific hybrids (Figure 1E–F). Even more unexpected, we observe that TE families derepressed in F1 hybrids exhibit lower silent divergence (*Ks*) between species than nonderepressed TEs (Figure 2A). These results demonstrate that mismatches between piRNAs and TE transcripts cannot explain the pattern of TE derepression in hybrids.

### TEs Derepressed in Interspecific Hybrids Are Enriched for Transpositionally Active and Horizontally Transferred Families

In *Drosophila*, endogenous TEs exhibit an exceptionally high rate of horizontal transfer between the genomes of divergent species [9],[10],[12]. A recent genome-wide analysis estimates that as many as 40% of TE families in the *D. melanogaster* genome and 36% of TE families in the *D. simulans* genome were involved in recent horizontal transfers, based on their exceptional sequence similarity to a TE family in a different *Drosophila* species [10]. Therefore, the relatively low *Ks* values of TEs derepressed in interspecific hybrids suggest that they are enriched for families that have been recently horizontally transferred (Figure 2A). Of the 32 TE families derepressed in hybrids, 12 (of 19 examined) were identified previously as candidates for horizontal transfer between *D. melanogaster* and *D. simulans* (Figure 1A) [10],[12]. An additional TE family, *mdg3*, is a candidate for horizontal transfer between the *D. melanogaster* and *D. yakuba* genomes [10]. Collectively, therefore, TEs derepressed in interspecific hybrids are enriched for candidate horizontally transferred families, relative to those that remain silenced (*G-test* of independence = 3.912, *p* = 0.048).

Because piRNA-mediated silencing is dependent on base complementarity, the observation that TE families that are virtually identical in sequence between *D. melanogaster* and *D. simulans* become derepressed in their interspecific hybrids is highly unexpected. One potential explanation for this result is that horizontally transferred TEs are among those families that are most transcriptionally active and therefore are exceptionally sensitive to disruptions in piRNA-mediated silencing. Indeed, we found that TE families derepressed in interspecific hybrids show elevated transcript abundance in the ovaries of both parental pure species (Figure 2B–C and Figure S2). Derepressed TE families furthermore exhibit an elevated proportion of polymorphic insertions segregating at very low frequency (∼<1.5%) in natural populations of *D. melanogaster* [52],[53], suggesting that they exhibit high transposition rates (Figure 2D).

### Interspecific Hybrids Phenocopy piRNA-Effector-Protein Mutants in Their Pattern of TE Derepression

**Figure 3:**
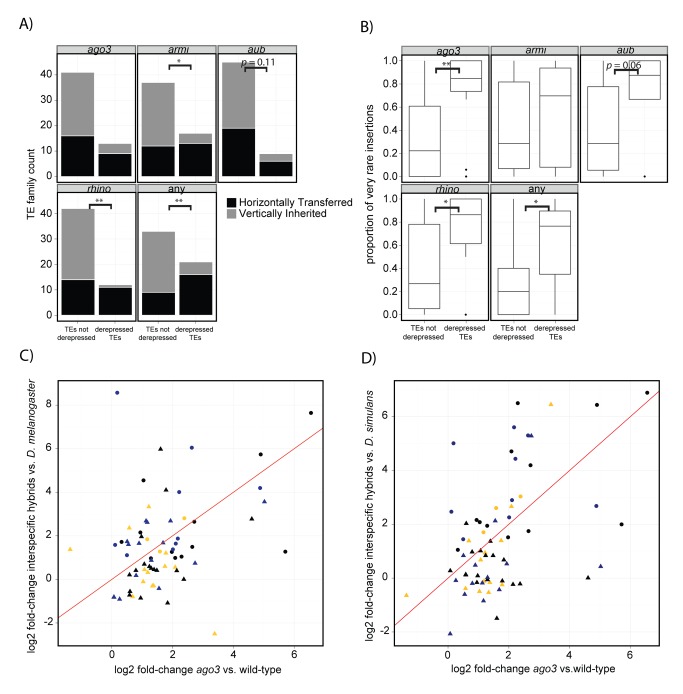
TE activity in interspecific hybrids phenocopies piRNA pathway mutants. (A) TE families derepressed in four piRNA pathway mutants are enriched for candidates for recent horizontal transfer [10],[12]. Significance of comparisons between derepressed TE families and nonderepressed TE families was determined by a *G-*test of independence. “Any” category considers all TE families derepressed in one or more of the four mutant backgrounds. Derepressed TE families are from [44],[45]. (B) TE families derepressed in four piRNA pathway mutants exhibit a higher proportion of insertions segregating at very low frequency [52],[53]. Statistical significance was assessed by a Wilcoxon Rank Sum Test. (C and D) Changes in TE activity in interspecific hybrids versus *D. melanogaster* (C) and *D. simulans* (D) correlate with changes in TE activity in *ago3* mutants versus wild-type *D. melanogaster* [45]. Color of TE classes is from Figure 1C and suggests that these correlations are independent of interspecific differences in the piRNA pool. Circles and triangles represent TE families derepressed and nonderepressed, respectively, in interspecific hybrids (Figure 1A). Red line indicates the best fit regression line. * *p*<0.05. ** *p*<0.01. *** *p*<0.001.

If the derepression of horizontally transferred, transpositionally active TE families in interspecific hybrids is indicative of disrupted piRNA-mediated TE silencing, we expect these same TE families to become derepressed in piRNA-effector-protein mutants. Consistent with this prediction, we discovered from published data that TEs derepressed in any of four germline piRNA-effector protein mutant backgrounds, *ago3*, *armitage* (*armi*), *aub*, or *rhino* (*rhi*), are enriched for candidates of recent horizontal transfer (Figure 3A). We consider TE families derepressed in any piRNA-effector-protein mutant background in order to identify general characteristics of TE families regulated by piRNA-mediated silencing. However, all mutant backgrounds also individually exhibit an elevated abundance of horizontally transferred TE families among those that are derepressed, and for *armi* and *rhi* mutants, this represents a significant enrichment (Figure 3A). Similarly, TEs derepressed in any of four piRNA pathway mutant backgrounds exhibit a significantly higher proportion of polymorphic insertions segregating at very low frequency in natural populations of *D. melanogaster* compared to nonderepressed TE families, and *ago3* and *rhi* mutants individually exhibit a significantly higher proportion of very rare insertions among derepressed TE families (Figure 3B).

Interspecific hybrids further resemble piRNA-effector-protein mutants in their quantitative profile of TE derepression. For individual TE families, changes in transcript abundance in interspecific hybrids, relative to their parental species, are significantly correlated with changes in transcript abundance observed in all four piRNA mutant backgrounds, relative to wild-type flies (Figure 3C–D and Figure S3). The strongest among these correlations is between hybrids and *argonaute-3* (*ago3*) mutants (hybrids/*D. simulans* Pearson's *r* = 0.49, *p* = 1.9×10−^5^; hybrids/*D. melanogaster* Pearson's *r* = 0.37, *p* = 0.0016), although partial correlations suggest that hybrid TE activity mostly parallels variation common to all germline piRNA mutant backgrounds (Table S4). Because our measurements of TE activity used different platforms (RNA-seq versus microarrays) and different annotations than those used for studies of piRNA-effector protein mutants, our findings likely underestimate the true extent to which TE activity in interspecific hybrids mirrors that observed in piRNA mutant backgrounds.

### Interspecific Hybrids Phenocopy piRNA-Pathway Mutants in Deficient piRNA Production and Aberrant piRNA Cluster Activity

**Figure 4:**
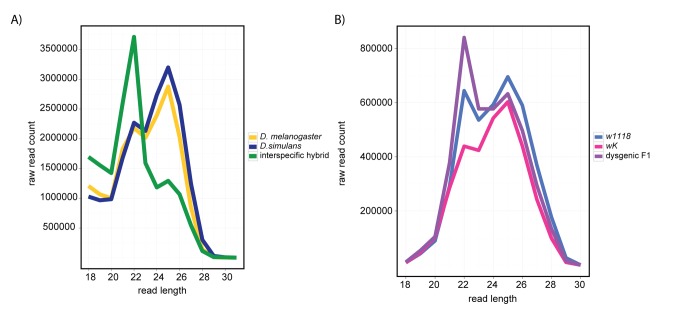
Small RNA pools from interspecific hybrids, but not intraspecific hybrids, are depauperate for (23–29 nt) piRNAs. (A) Size distribution of small RNAs from *D. melanogaster* (yellow), *D. simulans* (blue), and interspecific hybrid (green) ovaries. (B) Size distribution of small RNAs from *w^K^* (pink), *w^1118^* (blue), and their F1 dysgenic intraspecific hybrid (purple) ovaries.

In piRNA-effector-protein mutants, TE derepression frequently is associated with multiple defects in piRNA production. To determine if interspecific hybrids also experience problems making piRNAs, we applied deep sequencing to their ovarian small RNA pool (18–30 nt). Many piRNA pathway mutants are characterized by decreased piRNA abundance (≥23 nt), which shifts the size distribution of ovarian piRNAs towards miRNAs and endo-siRNAs (18–22 nt) [18],[41]. Strikingly, we observed this exact phenotype in interspecific hybrid ovaries, whereas the ovaries of parental pure species exhibit abundant piRNAs (Figure 4A). By contrast, the size profile of small RNAs from intraspecific *I-element* dysgenic ovaries is not dramatically skewed, indicating that piRNA loss is not a general feature of hybrid dysgenesis (Figure 4B).

Many piRNAs originate from precursor transcripts of TE-rich piRNA clusters. In piRNA-effector-protein mutants, the contribution of individual piRNA clusters to the piRNA pool can be altered dramatically [18],[44]. We determined the abundance of piRNAs derived from the 15 most prolific piRNA clusters in the *D. melanogaster* genome [17] in the ovarian piRNAs of interspecific hybrids and their parental pure species. piRNA clusters are absent or incompletely assembled in the *D. simulans* published genome [30], preventing an equivalent analysis of paternal piRNA clusters.

**Figure 5:**
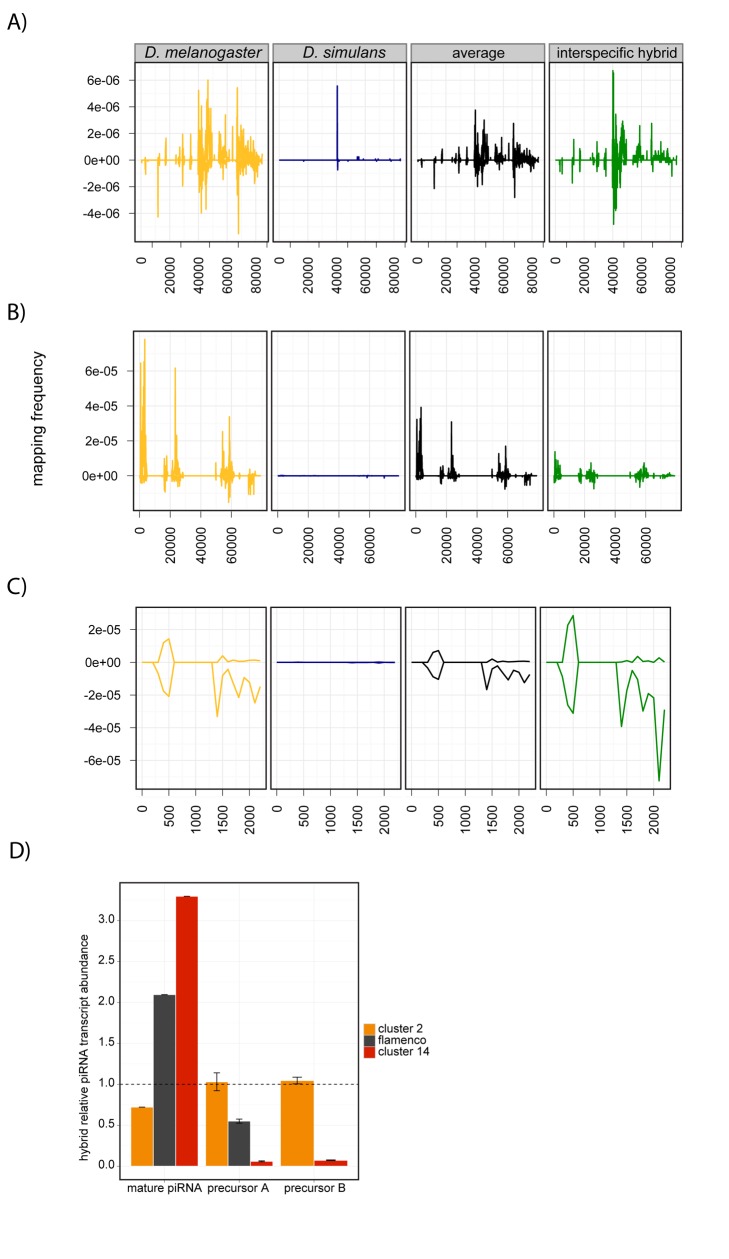
Aberrant activity of piRNA clusters in interspecific hybrids. (A–C) Frequency of uniquely mapping piRNAs derived from *D. melanogaster* piRNA clusters 9 (A), 5 (B), and 10 (C) for *D. melanogaster* (yellow), *D. simulans* (blue), and interspecific hybrids (green). Average indicates the additive expectation for interspecific hybrids (black). (D) Abundance of precursor transcripts (qRT-PCR) and mature piRNAs (small RNA-seq) from piRNA clusters 2, 14, and *flamenco*, relative to the interspecific average. Dotted line indicates no change in relative expression. Precursors A and B denote two independently measured locations in the precursor transcript.

The expected abundance of piRNAs derived from an individual cluster in hybrid ovaries is the interspecific average of their abundance in the ovaries of *D. melanogaster* and *D. simulans*. Five piRNA clusters conform to this additive expectation (Figure 5A, Figure S4, Table S5). Five other clusters exhibit underdominant inheritance, as their activity is well below our additive expectation (Figure 5B, Figure S4, Table S5). The remaining five exhibit overdominant inheritance in interspecific hybrids, with activity levels well above the level of their *D. melanogaster* mothers and more than 2-fold greater than the interspecific average (Figure 5C, Figure S4, Table S5). Again, this phenotype differs from that of intraspecific hybrids, where only a single piRNA cluster (Cluster 7) does not exhibit additive inheritance (Table S6).

Unusual cluster activity could arise either from aberrations in primary transcription or downstream processing. RT-PCR of primary transcripts from four piRNA clusters revealed that transcript abundance ranges from wild-type to severely reduced (Figure 5D, Figure S5). We furthermore detected an inverse relationship between the abundance of precursor transcripts and mature piRNAs: High relative abundance of the precursor transcript is associated with low relative abundance of the mature piRNAs, and vice versa (Figure 5D). This pattern of accumulated precursor transcript and reduced numbers of mature piRNAs likely indicates a defect in processing of precursor piRNA transcripts [21]. Our data in total suggest that hybrids suffer from at least two distinct defects in piRNA production from piRNA clusters.

### Interspecific Hybrids Exhibit Deficient Ping Pong Amplification and Mislocalization of Aubergine and Ago3

For many TE classes, the “ping pong” amplification loop is postulated to process precursor transcripts into mature piRNAs [17],[54]. This cycle produces both sense and anti-sense piRNAs and is mediated by two Piwi-clade Argonaute proteins, Aub and Ago3 (reviewed in [13]). Aub- and Ago3-mediated cleavage preferentially occurs 10 bp offset from the 5′ end of the complexed piRNA [54]. The robustness of ping pong amplification, therefore, is indicated by the ping pong fraction, or the probability that a randomly sampled piRNA from a TE family will have a complementary binding partner whose 5′ end is 10 bp offset.

**Figure 6:**
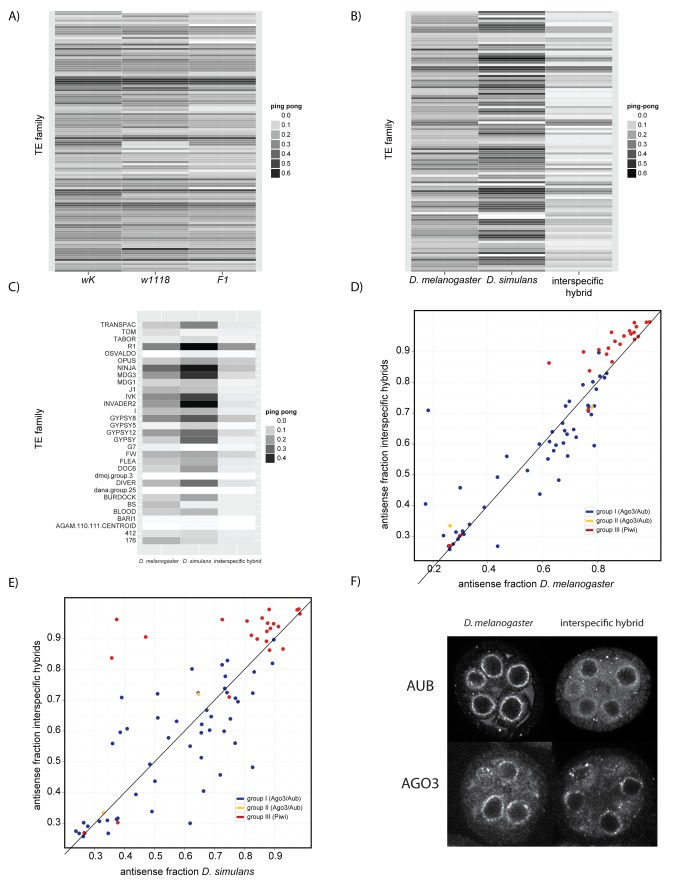
Interspecific hybrids phenocopy piRNA pathway mutants in disrupted piRNA production and nuage mislocalization. (A and B) Aberrant ping pong fractions in interspecific but not intraspecific hybrids. Ping pong fractions [23] for all TE families are compared between intraspecific hybrids and their parents (A) and interspecific hybrids and their parents (B). Only TE families that were represented by >50 small RNA reads and a ping pong fraction >0.1 in at least one of the three libraries are shown. (C) Ping pong fractions for TE classes derepressed in interspecific hybrids (taken from Figure 1A). (D and E) Increased antisense fraction of group III elements in interspecific hybrids. The antisense fraction of piRNAs derived from individual TE families in interspecific hybrids compared to *D. melanogaster* (D) and *D. simulans* (E). Classification of TE groups is from Li et al. [45]. Black line indicates equivalent antisense fractions in interspecific hybrids and parental pure species. (F) Anti-Aub and anti-Ago3 staining in *D. melanogaster* and interspecific hybrids in stage 2–6 egg-chambers. anti-Ago3 cross-reacts with *D. simulans*, but anti-Aub does not. Scale bars: 10 µm.

Although intraspecific hybrids exhibit no evidence for disrupted ping pong amplification when compared to their parental strains, the ping pong fraction for most TE families was reduced in interspecific hybrids, suggesting global dysfunction of the ping pong amplification loop (Figure 6A–B). We furthermore observed that for many TE families, the ping pong signature was more robust in *D. simulans* than in *D. melanogaster* (Figure 6B). Because ping pong amplification does not differ significantly between *D. melanogaster* (*Hmr*/+) and other wild-type *D. melanogaster* strains (Figure S1B), this may represent an interspecific difference in the robustness of ping pong amplification.

Interestingly, TE derepression in interspecific hybrids is not always associated with disrupted ping pong amplification, because the ping-pong fraction is not exceptionally reduced in misregulated TEs when compared to all TE families (Figure 6C). Furthermore, eight derepressed TE classes exhibit no evidence for ping pong processing in one or both parental species, suggesting that they are not dependent on the ping pong amplification loop for piRNA production and associated TE silencing (Figure 6C). Similar results have been observed in mutants of piRNA-effector proteins involved in the ping pong cycle, with TE families such as *blood* and *412* becoming misregulated despite not having a robust ping pong fraction in wild type ovaries [44],[45]. Our observations, therefore, are consistent with the disrupted function of effector proteins involved in ping pong amplification in the hybrid genetic background.

A second outcome of the ping pong amplification loop is that it produces both sense and antisense piRNAs in a ratio specific to each TE family [45]. In *aub* and *ago3* mutant backgrounds, for example, the antisense fraction of Aub/Ago3-regulated TE classes decreases, whereas for Piwi-regulated elements, the antisense fraction increases slightly [45]. When comparing interspecific hybrids to their parental pure species, we similarly observe that Piwi-regulated elements experience a disproportionate increase in their antisense fraction when compared to Aub/Ago3-regulated elements (Figure 6D–E; *D. melanogaster*, *t* = 4.044, *df* = 71, *p* = 1.32×10^−4^; *D. simulans*, *t* = 5.043, *df* = 71, *p* = 3.38×10^−6^), reinforcing our inference of disrupted ping pong processing.

Many piRNA proteins, including Aub and Ago3, localize to the nuage, a germline-specific perinuclear organelle [17],[42],[43],[55],[56]. Ping pong amplification is thought to occur in the nuage, and most piRNA mutant backgrounds that affect ping pong amplification also exhibit mislocalization of some or all nuage proteins [18],[44],[45]. In interspecific hybrids, Ago3 and Aub are partially dispersed to the cytoplasm (Figure 6F), consistent with the disruption in ping pong amplification we observed in these ovaries (Figure 6B). Intriguingly, however, other nuage components such as Vasa and Krimper, which are mislocalized in *aub* or *ago3* mutant backgrounds [43],[45], are not mislocalized in hybrids (Figure S6), suggesting incomplete disruption of nuage in the hybrid genetic background.

### Functional Divergence between *D. melanogaster* and *D. simulans* in the Key Piwi-Argonaute Protein Aubergine

The striking similarities between interspecific hybrids and piRNA-effector-protein mutants, both in their profiles of TE derepression and their deficiencies in piRNA production, suggest that piRNA-effector proteins may not function optimally in hybrid ovaries. An appealing explanation for these observations is that piRNA pathway dysfunction is a consequence of adaptive divergence in multiple piRNA-effector proteins between *D. melanogaster* and *D. simulans* [30]–[33]. For example, *aubergine* exhibits a highly significant excess of nonsynonymous substitutions between *D. melanogaster* and *D. simulans*, suggesting it has experienced strong directional selection since the divergence of these two lineages [30],[32],[33].

**Figure 7:**
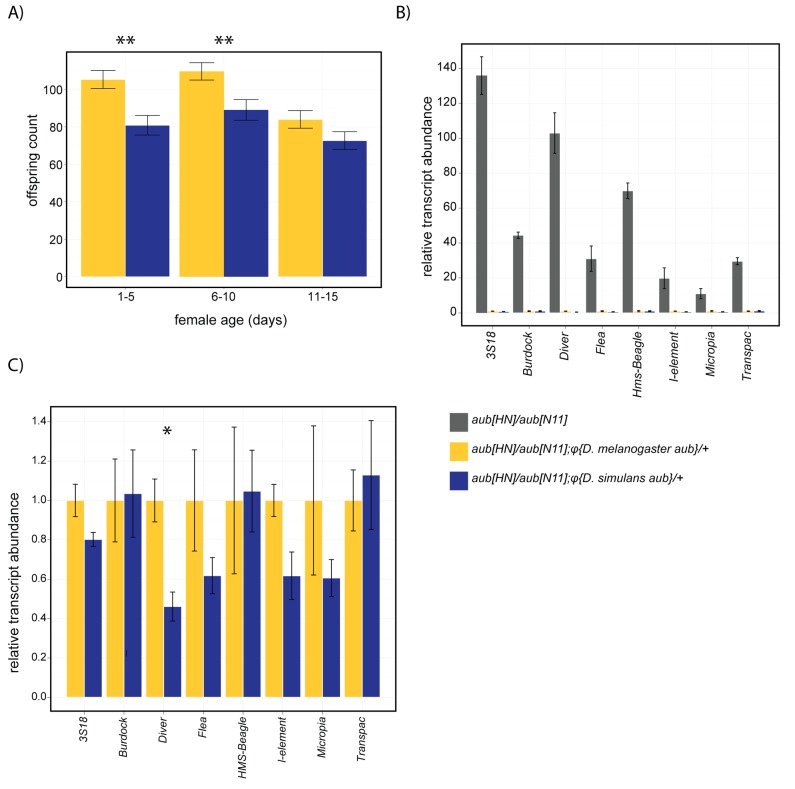
*D. simulans aubergine* does not fully complement a *D. melanogaster aubergine* mutant. φC31-mediated integrations of *D. simulans aubergine* and *D. melanogaster aubergine* transgenes are compared for their ability to complement *aub^HN^/aub^N11^* for (A) female sterility across three different age ranges and for TE transcript abundance (B and C). Panel C contains the same data from the transgenic genotypes as in (B), replotted on a larger scale to illustrate potential differences between them. TE transcript abundance was determined relative to *rpl32* and is scaled to the transcript abundance in *aub^HN^/aub^N11^;φ{D. melanogaster aubergine}/+.*

To determine whether adaptive evolution is associated with diverged protein function, we examined the ability of *D. simulans aubergine* to complement a *D. melanogaster aubergine* mutant (Figure 7). By comparing site-specific integrations of genomic transgenes that we determined do not differ in expression levels (*p* = 0.11; Figure S7), we ensured that functional differences between *D. melanogaster* and *D. simulans* alleles are attributable to their coding sequences. Although both transgenes complemented the complete female sterility of trans-heterozygotes, the *D. simulans* transgene exhibited lower fertility across a time-course (*F_1,136_* = 8.06, *p* = 0.0052; Figure 7A), with significant differences between transgenes for both 1–5- (*t_128_* = 3.2042, *p* = 0.0017) and 6–10-d-old females (*t_120_* = 2.6342, *p* = 0.0095, Figure 7A). Thus, *D. simulans aubergine* is not equivalent to its *D. melanogaster* ortholog when functioning in a *D. melanogaster* background.

To determine if reduced fertility is associated with broad TE derepression, we compared the ability of the two transgenes to regulate eight TE families previously described as derepressed in *aubergine* mutant backgrounds [45], of which five are also derepressed in interspecific hybrids (Figure 7B). Surprisingly, we observed that both transgenes provide essentially equivalent complementation (Figure 7B), with only one TE family, *Diver*, differing significantly in expression between them (*F_1,4_* = 18.77, *p* = 0.012; Figure 7C). Functional divergence of *aubergine* alone, therefore, cannot explain TE derepression in the interspecific hybrids. Instead, we suggest that TE derepression reflects the accumulated divergence of multiple piRNA regulatory genes.

## Discussion

### Interspecific Hybrids Phenocopy piRNA-Effector-Protein Mutations

The absence of an association between TE activity in F1 hybrids and interspecific differences in ovarian piRNAs suggests that TE derepression in interspecific hybrids does not result predominantly from a deficiency of maternally deposited piRNAs. Rather, we demonstrated that interspecific hybrids recapitulate the major phenotypes of piRNA-effector-protein mutants. These phenotypes include global derepression of recently active and candidate horizontally transferred TE families and a dramatic loss of piRNAs that is associated with specific defects in the transcription and processing of precursor piRNAs.

The close phenotypic resemblance between piRNA pathway mutants and interspecific hybrids suggests that the function of piRNA-effector proteins is disrupted or aberrant in the hybrid genetic background. In particular, the pattern of TE derepression, reduced ping pong amplification, and disrupted localization of nuage components that we observe in interspecific hybrids most closely phenocopies components of the *Aubergine* (*aub*)/*Argonaute-3* (*ago3*)-dependent germline piRNA pathway [18],[44],[45]. It is impossible to attribute the hybrid phenotype to the dysfunction of specific piRNA-effector proteins, however, because this suite of defects is common to many piRNA-effector protein mutant backgrounds, including *aub*, *ago3*, *krimp*, *rhino*, *spnE*, *vasa*, and *tejas* [18],[43]–[45],[57].

We argue below that hybrid dysfunction is due to divergence in the coding sequences of piRNA-effector genes. Although we cannot exclude some additional contribution from gene misexpression in hybrids, we found that most piRNA-effector proteins are similar in expression level between hybrids and their parental species. One effector gene, *BoYb*, has reduced transcript abundance in hybrid ovaries (25%–36% of wild-type levels). However, even a complete loss of *BoYb* function could not explain the complex phenotypes we observe in interspecific hybrid ovaries [39],[58]. First, *BoYb* functions in the germline piRNA pathway, but we observe misregulation in hybrids of somatic TE families such as *tabor*, *gypsy,* and *gypsy5* (Figure 1A, Table S3) [39]. Second, *BoYb* is partially functionally redundant with its paralog *Sister of Yb* (*SoYb*) [39], which has a wild-type expression level in interspecific hybrids (Table S2). Although the role of these proteins in the ping pong–dependent piRNA pathway is unclear, only the knockdown of both *BoYb* and *SoYb* together could lead to phenotypes such as the mislocalization of Aub and Ago3 or the dramatic derepression of *blood* (∼64-fold) [39],[58].

### Adaptive Protein Divergence as a Cause of TE Misexpression in Interspecific Hybrids

Interspecific hybrids often are characterized by deleterious phenotypes arising from the failure of two or more loci from the parental species to function optimally together. These incompatibility loci often correspond to protein-coding genes that have diverged between species by adaptive evolution (reviewed in [59],[60]). Many protein components of the *Drosophila* piRNA pathway similarly exhibit signatures of adaptive divergence between *D. melanogaster* and *D. simulans* [30],[32],[33], suggesting that they could contribute to these types of functional incompatibilities in interspecific hybrids.

Our interspecific complementation experiments with *aubergine* (*aub*) demonstrated that adaptive evolution of a piRNA protein can result in functional divergence, because *D. simulans aub* does not fully complement the female sterility caused by the loss of *aub* function in *D. melanogaster*. The replacement of *D. melanogaster aub* by *D. simulans aub* is not associated with dramatic TE derepression, however, indicating that functional divergence in *aub* alone cannot explain the complex phenotype of interspecific hybrids. Rather, the failure of *D. simulans aub* to fully complement when compared to its *D. melanogaster* counterpart suggests a mild incompatibility.

Similar to Aub, 10 other piRNA-effector proteins (Ago3, Yb, Armi, SpnE, Mael, Vasa, Piwi, Krimper, Tudor, and Kumo/Qin) exhibit an excess of amino acid changes between *D. melanogaster* and *D. simulans* [30],[32],[33]. Our results, therefore, are consistent with a model in which the cumulative effect of this divergence among multiple genes results in the dramatic piRNA pathway dysfunction we observed in interspecific hybrids. Interspecific complementation offers a promising approach to assay diverged functions of individual piRNA pathway components in future studies.

### Contrasting Properties of TE Misexpression in Interspecific and Intraspecific Hybrids

Intraspecific hybrid dysgenesis provides an important comparison for interpreting our interspecific data. While the parents of these dysgenic offspring differ in terms of their ovarian piRNA pools, particularly in the abundance of the dysgenic element, they do not exhibit interspecific divergence in their encoded piRNA proteins. Thus, if piRNA-mediated silencing were generally disrupted in intraspecific dysgenesis, it would argue against our interpretation of a protein-mediated incompatibility causing widespread TE misregulation in interspecific hybrids.

The dysgenesis syndrome we observed in *D. melanogaster* intraspecific hybrids contrasts with that of interspecific hybrids in several important respects. In interspecific hybrids, we observed ≥2-fold increased transcript abundance in 32 unique TE families when compared to both *D. melanogaster* and *D. simulans* pure species (Figure 1A). We furthermore observed that these changes in transcript abundance are highly correlated with those observed in piRNA-effector protein mutants (Figure 3C–D). In contrast, intraspecific *I-*element dysgenesis is characterized by only 11 TE families that exhibit increased transcript abundance when compared to either the maternal or the paternal strain, but not both (Figure 1B).

Many TE families are variable in copy number within and between species and presumably also differ in total expression level. A simple null hypothesis therefore is that TE expression level in either intraspecific or interspecific hybrids will be additive—that is, the average of the parental strains. If TE expression is much higher in the paternal strain relative to the maternal strain, then one also expects a higher expression level in the hybrids relative to the maternal strain, even in the absence of transcriptional derepression. Our observations of TE transcript abundance in *I-*element dysgenic ovaries are consistent with this scenario of intraspecific polymorphism in TE copy number or expression. In light of these findings, we suggest that a conservative definition of transcriptional “TE derepression” is that the TE family is significantly overexpressed relative to both parental strains.

Intraspecific and interspecific hybrids further differ with respect to the role of piRNA production in TE regulation. *I-*element dysgenesis is specifically associated with a paucity of *I-*element-derived piRNAs in the ovaries of the maternal cytotype [23],[24]. We found that piRNA production appears to be otherwise normal from *I-*element dysgenic ovaries, demonstrating that intraspecific dysgenesis does not impact the overall function of the piRNA pathway (Figures 4B, 6A, and Table S6). These data therefore fit a model in which interstrain differences in *I-*element-derived piRNAs explains *I-*element dysgensis. In contrast, TEs derepressed in interspecific hybrids exhibit no relationship to interspecific differences in the ovarian piRNA pool (Figure 1D). Rather interspecific hybrids exhibit a general loss of piRNAs that is characteristic of mutant backgrounds that disrupt piRNA production (Figure 4A).

### Does Adaptive Evolution of piRNA-Effector Proteins Contribute to Interspecific Hybrid Sterility and Speciation?

The observation that TEs can spread rapidly through populations and cause sterility when infected populations interbreed with uninfected populations led to suggestions that TEs may cause the rapid evolution of reproduction isolation [26]. This idea fell into disfavor as studies indicated that *Drosophila* interspecific hybrids from three distinct species pairs do not exhibit hallmarks of TE derepression, such as elevated mutation or recombination rates [61],[62]. Furthermore, complete sterility is a transient developmental state in many intraspecific hybrid dysgenesis systems [20],[63],[64], and recent studies suggest that host genome resistance evolves rapidly by the incorporation of representative TEs into piRNA clusters and the maternally deposited piRNA pool [19],[20],[29]. Thus, it seems unlikely that differences in active TE families could provide a stable barrier to gene flow between diverging lineages.

Although we did not address what impact TE derepression has on the fecundity of *D. melanogaster/D. simulans* hybrids, our results suggest that the accumulated divergence of piRNA-effector proteins may contribute to reduced fertility in interspecific hybrids. Furthermore, rapidly evolving heterochromatin and heterochromatin proteins can cause hybrid incompatibility in *Drosophila* [65]–[67]. Because heterochromatin formation is implicated in TE silencing, heterochromatin divergence may also contribute to the TE derepression that we observe in interspecific hybrid ovaries. Taken together, these findings suggest an alternative view, in which TEs contribute to hybrid incompatibility but indirectly through their evolutionary impact on host gene evolution, which in turn leads to TE depression. Interestingly, TE derepression has been observed in the F1 interspecific hybrids of tammar and swamp wallabies [68], in F1 allopolyploid hybrids from crosses between closely related *Arabidopsis* species [69], and between divergent wheat genera [70]. Although the contribution of protein divergence remains unknown in these examples, the wheat hybrids suffer a loss of TE-derived small RNAs, similar to what we describe in *Drosophila* interspecific hybrids [70].

### What Is Driving Adaptive Evolution of piRNA-Effector Proteins?

While the underlying selective forces await discovery, TEs are obvious candidates to drive adaptive evolution of piRNA-effector proteins. TE activity can change dramatically over short evolutionary time scales, and many TEs in the *Drosophila melanogaster* genome exhibit evidence of recent transpositional bursts [71],[72]. Furthermore, the exceptional sequence similarity of TEs from divergent lineages suggests that genomes frequently are invaded by novel, horizontally transferred TEs [9]–[12]. Consistent with a role for piRNA-effector proteins in host genome defense, natural genetic variation in the nuage component Vasa has been associated with variable suppression of the LTR-retrotransposon *copia* [73].

Antagonistic coevolution between TEs and the piRNA pathway could be analogous to that occurring between viruses and the siRNA pathway, in which host effector-proteins must adapt to avoid functional disruption by virally encoded proteins [74]–[76]. Alternatively, piRNA proteins may evolve rapidly in response to changes in the content or distribution of the genomic TE pool. Regardless of the underlying mechanism, our study reveals the significant consequences of the evolutionary interplay between piRNA-effector proteins and the dynamic population of TEs that inhabit eukaryotic genomes.

## Materials and Methods

### Generation of Transgenic Lines

Primers aub-F and aub-melR/aub-simR were used together with iProof high-fidelity taq DNA polymerase (Bio-Rad) to amplify the ∼7 Kb *aubergine* genomic region from *D. melanogaster y^1^; cn^1^ bw^1^ sp^1^* and *D. simulans w^501^* (*D. melanogaster* Release 5, 2L:11003713..10996581; *D. simulans* Release 1, 2L: 10801253..10797868). This region includes ∼2 Kb of upstream sequence to ensure the presence of the endogenous regulatory elements [77]. The resultant PCR fragments were cloned into pCR-Blunt-II-Topo according to manufacturer instructions (Invitrogen), and the inserts of both plasmids were verified by sequencing to be free of mutations. The p{*D. melanogaster aub*} and p{*D. simulans aub*} plasmids were generated by subcloning the NotI/SpeI fragment of each TOPO plasmid into NotI/XbaI-linearized pCasper4/attB [78].

φC31-mediated transformation was used to introduce p{*D. melanogaster aub*} and p{*D. simulans aub*} into *D. melanogaster* at the p(Cary)attP2 site [79] by Genetics Services Inc. (Cambridge, MA). Site-specific integration of both transgenes was verified by the PCR-method of Venken et al. [80]. The resulting transgenes (*φ{D. melanogaster aub}* and *φ{D. simulans aub}*) were made homozygous in *D. melanogaster w^1118^* and then crossed into *yw; aub^HN^/CyO* [77].

### *Drosophila* Stocks, Rearing, and Crosses

For interspecific hybrid dysgenesis assays, *D. melanogaster* females (*In(1)AB, Hmr^2^*/*FM6*), *D. simulans* females (*w^501^*), and F1 interspecific hybrid females from the cross of *In(1)AB, Hmr^2^*/*FM6* females to *w^501^* males were collected as virgins from crosses at 18°C and reared at 18°C on standard cornmeal media, supplemented with yeast to enhance oogenesis. For intraspecific hybrid dysgenesis assays, *D. melanogaster w^K^* females, *w^1118^* females, and F1 hybrid females (*w^K^*×*w^1118^*) were collected as virgins from crosses at 22°C and reared at 22°C.

For immunocytochemistry and Illumina small RNA and mRNA library construction from interspecific hybrids and their parental pure species, ovaries from 4–6-d-old females were used. For Illumina mRNA library construction of intraspecific hybrids and their parental strains, ovaries from 2–4-d-old ovaries were used. For qRT-PCR of TE transcript abundance in transgenically rescued *aubergine* mutants, ovaries from 3–6-d-old females were used.

For interspecific complementation assays of female fertility, virgin females *w; aub^N11^ bw^1^/CyO* were crossed to *yw; aub^HN^ bw^1^/CyO; φ{D. melanogaster aub}/+* or *y w; aub^HN^ bw^1^/CyO; φ{D. simulans aub}/+* males, and crosses were maintained at 25°C. Virgin females (1) *w/y w; aub^HN^ bw^1^/aub^N11^ bw^1^; φ{D. melanogaster aub}/+*; (2) *w/yw; aub^HN^ bw^1^/aub^N11^ bw^1^; φ{D. simulans aub}/+*; and (3) *w/yw; aub^HN^ bw^1^/aub^N11^ bw^1^; +/+* were collected from both crosses and reared on standard cornmeal media with two males at 25°C. Fresh media and males were provided every 5 d. Females that did not produce offspring over the 15-d period were PCR tested for the presence of a transgene using the method of Venken et al. [80]. The PCR was necessary because the *w^+^* marker on the transgenes was difficult to score in a *bw^1^/bw^1^* background. All females that did not harbor a transgene were sterile.

For qRT-PCR, *aub^N11^ bw^1^/CyO* were crossed to (1) *yw; aub^HN^ bw^1^/CyO; +/+*; (2) *yw; aub^HN^ bw^1^/CyO; φ{D. melanogaster aub}/φ{D. melanogaster aub}*; or (3) *yw; aub^HN^ bw^1^/CyO; φ{D. simulans aub}/φ{D. simulans aub}* males, and crosses were maintained at 25°C. Virgin females (1) *w/yw; aub^HN^ bw^1^/aub^N11^ bw^1^; φ{D. melanogaster aub}/+*; (2) *w/yw; aub^HN^ bw^1^/aub^N11^ bw^1^; φ{D. simulans aub}/+*; and (3) *w/yw; aub^HN^ bw^1^/aub^N11^ bw^1^; +/+* were collected from these crosses and reared on standard cornmeal media supplemented with yeast paste at 25°C. Experimental females were maintained at a density of no greater than 20 flies per vial post-eclosion. Ovaries from 3–5-d-old females were used.

### Immunocyctochemistry

Dissected ovaries were fixed and stained according to [81]. Ovarioles were teased apart with insect pins, and tissue was fixed in a 100 mM cacodylate, 8% paraformaldehyde buffer. Primary antibody dilutions were 1∶10,000 (anti-Krimper, [43]), 1∶25 (anti-Vasa; Developmental Studies Hybridoma Bank), 1∶100 (anti-Maelstrom, [82]), or 1∶1000 (anti-Armi, [83]; anti-Aub, anti-Ago3, and anti-Piwi [17]). Prepared ovaries were visualized on a Zeiss 710 confocal microscope or a Leica SP2 confocal microscope. All antibodies were generated using *D. melanogaster* proteins; however, except for anti-Aub and anti-Vasa, all cross-react with *D. simulans* (unpublished data).

### Small RNA Library Preparation

Illumina small RNA libraries were prepared according to the protocol of Brennecke et al. [23]. Briefly, dissected ovaries were placed directly in Trizol reagent (Invitrogen), homogenized, and total RNA was extracted according to the manufacturer's instructions. Small RNAs were size fractionated on 12% polyacrylamide/urea gel, ligated directly to a 3′ small RNA cloning adaptor (Linker-1 from IDT [84]), and again purified on a 12% polyacrylamide/urea gel. Purified, 3′ ligated small RNAs were subsequently 5′ ligated to an Illumina adaptor (5′ACACUCUUUCCCUACACGACGCUCUUCCGAUC-3′) and again size fractionated and purified from a 12% polyarylamide/urea gel. Purified RNAs were reverse transcribed to cDNA using Superscript II (Invitrogen) and a primer to the 3′ cloning adaptor. First strand cDNA product was amplified using primers to both the 3′ and 5′ adaptors and purified from a 2% agarose gel. Details of small RNA library data analysis are described in Text S1, Figures S8–S11, and Tables S8–S10.

### mRNA Library Preparation

mRNA libraries were constructed according to the protocol of [85]. Total RNA was extracted from dissected ovaries as above, and mRNA was purified using poly-T Dynabeads (Invitrogen) according to the manufacturer's instructions. Isolated mRNA was fragmented using fragmentation buffer (Ambion), ethanol precipitated, and reverse-transcribed using Superscript II (Invitrogen) and random hexamer primers. Remaining RNA was digested with RNAseH, and second-strand synthesis was performed using DNA polymerase I (Promega). cDNA was purified on a MinElute column (Qiagen), repaired with End-IT DNA repair kit (Epicentre), A-tailed with Klenow enzyme (New England Biolabs), and ligated to Illumina adaptors. Ligated cDNA was gel purified with the MinElute gel purification kit (Qiagen), PCR amplified, and gel purified again. Details of mRNA library data analysis are described in Text S1, Figures S8–S11, and Tables S8–S10.

### Quantitative RT-PCR

RNA was purified from homogenized ovaries with Trizol reagent (Invitrogen) according to the manufacturer's instructions, treated with 2 µL of DNaseI (Promega) in a 100 µL reaction for 2 h at room temperature, and then secondarily purified with the RNeasy kit (Qiagen). Exactly 5 µg total RNA was synthesized to first strand cDNA with random hexamer primers with Superscript III (Invitrogen). For analysis of cluster 14 precursor, *aubergine*, and TE transcripts, the final cDNA product was diluted 1∶8 and used directly, whereas for *flamenco*, cluster 2, and cluster 5 precursors, cDNA product was purified on a Qiaquick column (Qiagen). These alternate methods to generating the starting cDNA were used because they gave the most robust standard curves for their respective primer pairs.

Transcript abundance was estimated by fluorescent intensity using 2× SYBR green supermix (BioRad) on a MyiQ lightcycler (BioRad). Ribosomal protein 32 was used as a standard control for comparing the relative abundance of piRNA precursor transcripts between treatments using the standard curve method. For comparisons between *D. melanogaster* and interspecific hybrids, diluted or purified cDNA from both *D. melanogaster* and interspecific hybrid samples was further diluted 1∶5 to generate three replicates of a 5-point standard curve for both *D. melanogaster* and interspecific hybrids, to confirm equivalent efficiencies of amplification in both genetic backgrounds for each primer pair (Table S7). The *D. melanogaster* standard curve was then used to estimate the starting transcript abundance for three replicates of an experimental sample. For comparisons between transgenically rescued and nonrescued *aub* mutants, diluted cDNA from *yw/w; aub^HN^ bw^1^/aub^N11^ bw^1^;φ{D. melanogaster aub}/+* was diluted 1∶5 to generate a standard curve. Experimental samples were measured at a dilution of 1∶25.

Cluster 5 precursor transcript was at such low abundance in interspecific hybrids that it was not possible to accurately estimate its relative expression on the light-cycler. We therefore confirmed its dramatically reduced abundance in interspecific hybrids using semi-quantitative PCR on cDNA samples of known concentration (500 ng/µL and 50 ng/µL in Figure S5). This approach was also used for the *flam*-B primers for the *flamenco* precursor transcript, as they yielded poor data on the light-cycler. Primer pairs are listed in Table S7.

For comparisons between interspecific hybrids and their parental pure species, all primers pairs do not amplify any product from *D. simulans* cDNA; therefore, relative transcript abundance of each precursor transcript in *D. simulans* is assumed to be 0. The additive expectation for hybrid expression, therefore, is 50% of the *D. melanogaster* expression level.

## Supporting Information

Figure S1 *In(1)AB, Hmr^2^* heterozygotes exhibit a wild-type piRNA pool. (A) The size distribution of cloned small RNAS from *In(1)AB, Hmr^2^*/*FM6* ovaries is similar to that of other wild-type strains. (B) The ping pong fraction [23] of TE families sampled in *In(1)AB, Hmr^2^/FM6* piRNAs is similar to that of other wild-type strains. (C and D) The frequency of reads mapping to the *42AB* and *flamenco* piRNA clusters is similar between *In(1)AB, Hmr^2^*/*FM6* and other wild-type strains. Wild-type small RNA libraries are from [23].
(TIF)

Figure S2 Positive relationship between normalized TE transcript abundance in the ovarian mRNAs of parental pure species and TE derepression in interspecific hybrids. Log2 TE-derived transcript abundance in *D. melanogaster* (A) and *D. simulans* (B) ovarian mRNAs is compared for TE families not derepressed and derepressed in interspecific hybrids. TE transcript abundance was normalized by library size and the length of the consensus sequence for each TE family. Derepressed TEs were those whose transcript abundance increased 2-fold or more in interspecific hybrids when compared to their parental pure species, regardless of whether this increase was statistically significant. TE families derepressed in interspecific hybrids showed a higher average TE transcript abundance in parental pure species than those that were not derepressed (* Wilcoxon Rank-Sum *p*<0.05). These comparisons complement those presented in the main text (Figure 2B–C), where TE families were considered derepressed only if the 2-fold or greater increase in transcript abundance represented a significant difference in expression between the hybrids and their parents (q-value<0.05). The requirement of a statistically significant increase in expression could bias towards TE families with higher transcript abundance becoming derepressed in interspecific hybrids, because these TEs have a higher read count and thus more power to reject the null hypothesis of no difference in expression. However, because differences in TE transcript abundance in parental pure species are robust, even in the absence of a requirement that that the 2-fold or greater increased expression in interspecific hybrids is statistically significant (A–B), we conclude that they are not an artifact of statistical power.
(TIF)

Figure S3 Correlations between TE activity in interspecific hybrids and piRNA pathway mutants. Colors denote TE classes more abundant among *D. melanogaster* piRNAs (yellow), *D. simulans* piRNAs (blue), or nondifferentially abundant between the piRNAs of these two species (black), from Figure 1C. Circles denote TE classes inferred as derepressed in interspecific hybrids, whereas triangles denote TEs not derepressed in interspecific hybrids (as in Figure 1A). Red line denotes equivalent changes in expression in hybrids relative to parental pure species when compared to piRNA mutants [44],[45] relative to wild-type controls (*w^1118^*). (A) Interspecific hybrid/*D. melanogaster* versus *aub*/*Oregon-R* (Pearson's *r* = 0.30, *p* = 0.01). (B) Interspecific hybrid/*D. simulans* versus *aub*/*Oregon-R* (Pearson's *r* = 0.27, *p* = 0.03). (C) Interspecific hybrid/*D. melanogaster* versus *armi*/*w^1118^* (Pearson's *r* = 0.29, *p* = 0.02). (D) Interspecific hybrid/*D. simulans* versus *armi*/*w^1118^* (Pearson's *r* = 0.35, *p* = 0.004). (E) Interspecific hybrid/*D. melanogaster* versus *rhi*/*w^1118^* (Pearson's *r* = 0.29, *p* = 0.02). (F) Interspecific hybrid/*D. simulans* versus *rhi*/*w^1118^* (Pearson's *r* = 0.35, *p* = 0.004). After accounting for the correlations among different piRNA mutants, only the correlation between hybrids/*D. simulans* and *ago3/*wild-type *D. melanogaster* remains significant (Pearson's *r* = 0.4, *p* = 0.0054, Table S4).
(TIF)

Figure S4 Proportions of piRNA reads mapping to 15 heterochromatic piRNA clusters described in Brennecke et al. [17]. Frequency of *D. melanogaster* (yellow), *D. simulans* (blue), and interspecific hybrid (green) piRNAs mapping with zero mismatches to the piRNA cluster are shown. An additive interspecific average also is shown (black) as a prediction for hybrid read mapping. Top, reads mapping uniquely to the cluster. Middle, all reads mapping to the cluster, normalized by the number of other genomic mapping locations with zero mismatches. Bottom, all reads mapping to the cluster.
(PDF)

Figure S5 Semi-quantitative RT-PCR of piRNA precursor transcripts. Precursor transcript was amplified in a 20 µL PCR using 5 µL of 500 ng/µL *D. melanogaster* cDNA (M), 500 ng/µL interspecific hybrid cDNA (H), 50 ng/µL *D. melanogaster* cDNA (M 1∶10), 50 ng/µL interspecific hybrid cDNA (H 1∶10), or negative control (NC) using (A) Cluster5A, (B) Cluster5B, (C) FlamB, and (D) Rpl32 primer pairs.
(TIF)

Figure S6 Nuage localization of piRNA proteins in interspecific hybrids. Interspecific hybrids were compared with their *D. melanogaster* mothers for the localization of 5 nuage components (grey scale). Merged images are colocalization between each protein examined (green) and Vasa protein (red), an additional nuage component. Scale bar: 10 µm.
(TIF)

Figure S7 Quantitative RT-PCR of *aubergine* in mutant and transgenic backgrounds. TE transcript abundance was determined relative to *rpl32* and is scaled to the transcript abundance in *aub[HN]/aub[N11];φ{D. melanogaster aubergine}/+.*
(TIF)

Figure S8 Identification of TE-derived mRNAs and piRNAs. Yellow indicates TE-derived reads unique to the *D. melanogaster* genome, blue indicates TE-derived reads unique to the *D. simulans* genome, and green indicates TE-derived reads that are found in either genome. (A) mRNAs mapped to a database of consensus TEs. (B) mRNAs mapped to all annotated TE insertions in both the *D. melanogaster* and *D. simulans* genomes. (C) piRNAs mapped to a database of consensus TEs. (D) piRNAs mapped to a database of all annotated TE insertions in both the *D. melanogaster* and *D. simulans* genomes. More piRNA and mRNA reads that are unique to the *D. simulans* genome are identified as TEs when the database of all annotated TEs is used.
(TIF)

Figure S9 Identifying TE-derived reads by mapping to a consensus sequence. (A) Venn diagram of overlap between TE classes identified as derepressed in interspecific hybrids using the insertion mapping and consensus mapping approaches. (B) TE classes identified as derepressed using the consensus mapping approach. (C) Relationship between interspecific divergence in piRNA abundance for individual TE classes and derepression of those TE classes, when TE-derived piRNAs are identified by mapping to a consensus sequence. TE classes were categorized as *D. melanogaster* biased, *D. simulans* biased, or nondifferentially abundant, based on their relative abundance in *D. melanogaster* and *D. simulans* piRNAs. The proportion of TE classes in each of these categories that are derepressed in interspecific hybrids is indicated by the area shaded in red.
(TIF)

Figure S10 Ping pong fraction [23] calculated for piRNA reads mapping to the *D. melanogaster* genome only (A), to the *D. simulans* genome only (B), and to both genomes (C). Interspecific hybrids are compared to their parental pure species, *D. melanogaster* and *D. simulans*.
(TIF)

Figure S11 Data reanalysis excluding cluster-derived piRNAs. (A) Only five TE classes exhibit significant change in abundance within *D. melanogaster* piRNAs when piRNAs uniquely mapping to heterochromatic clusters are excluded from the analysis. (B) Relationship between interspecific divergence in piRNA abundance for individual TE classes and derepression of those TE classes. TE classes were categorized as *D. melanogaster* biased, *D. simulans* biased, or nondifferentially abundant, based on their relative abundance in *D. melanogaster* and *D. simulans* piRNAs. The proportion of TE classes from each of these categories that are derepressed in interspecific hybrids is indicated by the area shaded in red. piRNAs uniquely mapping to *D. melanogaster* piRNA clusters were excluded from each sequencing library before analysis.
(TIF)

Table S1 Protein-coding genes that are underexpressed and overexpressed in interspecific hybrids. “FBgn” and “Symbol” denote the fly base gene id and symbol of the misexpressed gene. “log2FC” denotes log 2 transformed normalized fold-change between the indicated genotypes. “Significant” indicates whether the preceding comparison represents a significant difference in gene expression, q-value<0.05. “mel” indicates *D. melanogaster*, “sim” indicates *D. simulans*, and “hyb” indicates interspecific hybrid. “Overexpressed” indicates the gene exhibits a ≥2-fold increased expression in interspecific hybrids when compared to both parental pure species. “Underexpressed” indicates that the gene exhibits ≥2 fold decreased expression in interspecific hybrids when compared to both parental pure species.
(XLSX)

Table S2 Relative expression of piRNA effector protein genes in *Drosophila* interspecific hybrids. “FBgn” and “Symbol” denote the fly base gene id and symbol of the piRNA effector protein. “log2FC” denotes log 2 transformed normalized fold-change between the indicated genotypes. “Significant” indicates whether the preceding comparison represents a significant difference in gene expression, q-value<0.05. “Mel” indicates *D. melanogaster*, “sim” indicates *D. simulans*, and “hyb” indicates interspecific hybrid.
(XLSX)

Table S3 Class, genomic representation, tissue of regulation, and mode of regulation for TE families derepressed in interspecific hybrids. For “class,” LTR equals long terminal repeat, non_LTR equals non-long-terminal repeat, and TIR equals transcribed interspersed repeat. “Full length TE representation” indicates the presence of at least one full-length insertion from this TE family in both the *D. melanogaster* and the *D. simulans* genomes, in the *D. melanogaster* genome only, or in neither genome [10]. “Germline versus somatic” refers to the cell type where the majority of piRNAs derived from that family are thought to be produced [18]. “Ago3/Aub” dependency indicates the necessity of Ago3 and Aub function for the production of piRNAs derived from this TE family [45]. ^1^Elements whose genomic representation we verified using TE annotations for the *D. melanogaster* and *D. simulans* genomes [49].
(XLSX)

Table S4 Correlations and partial correlations in changes in TE transcript abundance between piRNA mutant backgrounds and interspecific hybrids. Pearson's R correlations (upper right) and partial correlations (lower left) between log2 transformed fold changes in TE transcript abundance are indicated between four piRNA mutant backgrounds relative to wild-type flies [44],[45] and interspecific hybrids relative to their parental pure species. hybrid/mel, interspecific hybrids relative to *D. melanogaster*; hybrid/sim, interspecific hybrids relative to *D. simulans.* Bold values indicate Pearson's *r* correlations that are significantly different from 0, or partial correlation values that are higher than 95% of those generated by 10,000 random permutations of the correlation matrix.
(XLSX)

Table S5 Relative expression of the 15 most active piRNA clusters in interspecific hybrid ovaries. Clusters were identified from Brennecke et al. [17]. Raw read counts and read counts normalized for differences in library size are reported for (A) uniquely mapping reads only, (B) multiply mapping reads normalized for number of mapping locations, and (C) all mappable reads. Relative expression is the ratio of the normalized expression in interspecific hybrids to the normalized interspecific average. Clusters with relative expression values ≥2 or ≤0.5 are defined as overexpressed (green) and underexpressed (red), respectively.
(XLSX)

Table S6 Relative expression of the 15 most active piRNA clusters in *I-element* dysgenic ovaries. Clusters were identified from Brennecke et al. [17]. Raw read counts and a read count normalized for differences in library size are reported for (A) uniquely mapping reads only, (B) multiply mapping reads normalized for number of mapping locations, and (C) all mappable reads. Relative expression is the ratio of the normalized expression in F1 dysgenic hybrid ovaries to the average of the two parental strains. Clusters with relative expression values ≥2 or ≤0.5 are defined as overexpressed (green) and underexpressed (red), respectively.
(XLSX)

Table S7 PCR primers. *R^2^* and efficiency are indicated for primer pairs used in quantitative RT-PCR. All primers are from our own design except when noted otherwise.
(XLSX)

Table S8 Redundant TE consensus sequences between repbase and piler-DF. All redundant TEs were treated as the same family. Assigned group name indicates the naming convention for the current study.
(XLSX)

Table S9 Raw counts of TE-derived reads from *D. melanogaster*, *D. simulans*, and interspecific hybrid mRNA and piRNA sequencing libraries. Counts are from mapping to all annotated insertions in the *D. melanogaster* and *D. simulans* genomes (Materials and Methods).
(XLSX)

Table S10 Read mapping of mRNA and small RNA Illumina sequencing libraries from interspecific hybrids and their parental pure species. For TE-derived mRNAs, the percentages are of the total mappable nonribosomal reads. For candidate miRNAs and piRNAs, percentages are of the total mappable reads. For TE-derived candidate piRNAs, the percentages are of the total candidate piRNAs.
(XLSX)

Text S1 Supplementary experimental materials and discussion.
(DOCX)

## Abbreviations

*ago3*: *argonaute-3*
*armi*: *armitage*
*aub*: *aubergine*
*BoYb*: *Brother of Yb*
non-LTR: non-long-terminal repeat
piRNA pathway: Piwi-interacing RNA pathway
*rhi*: *rhino*
*SoYb*: *Sister of Yb*
TE: transposable element
