# Supplementary figures and images for: Drosophila Interspecific Hybrids Phenocopy piRNA-Pathway Mutants
Source: PLoS Biol. 2012 Nov 20;10(11):e1001428. doi: 10.1371/journal.pbio.1001428 (PMC3506263; doi:10.1371/journal.pbio.1001428)

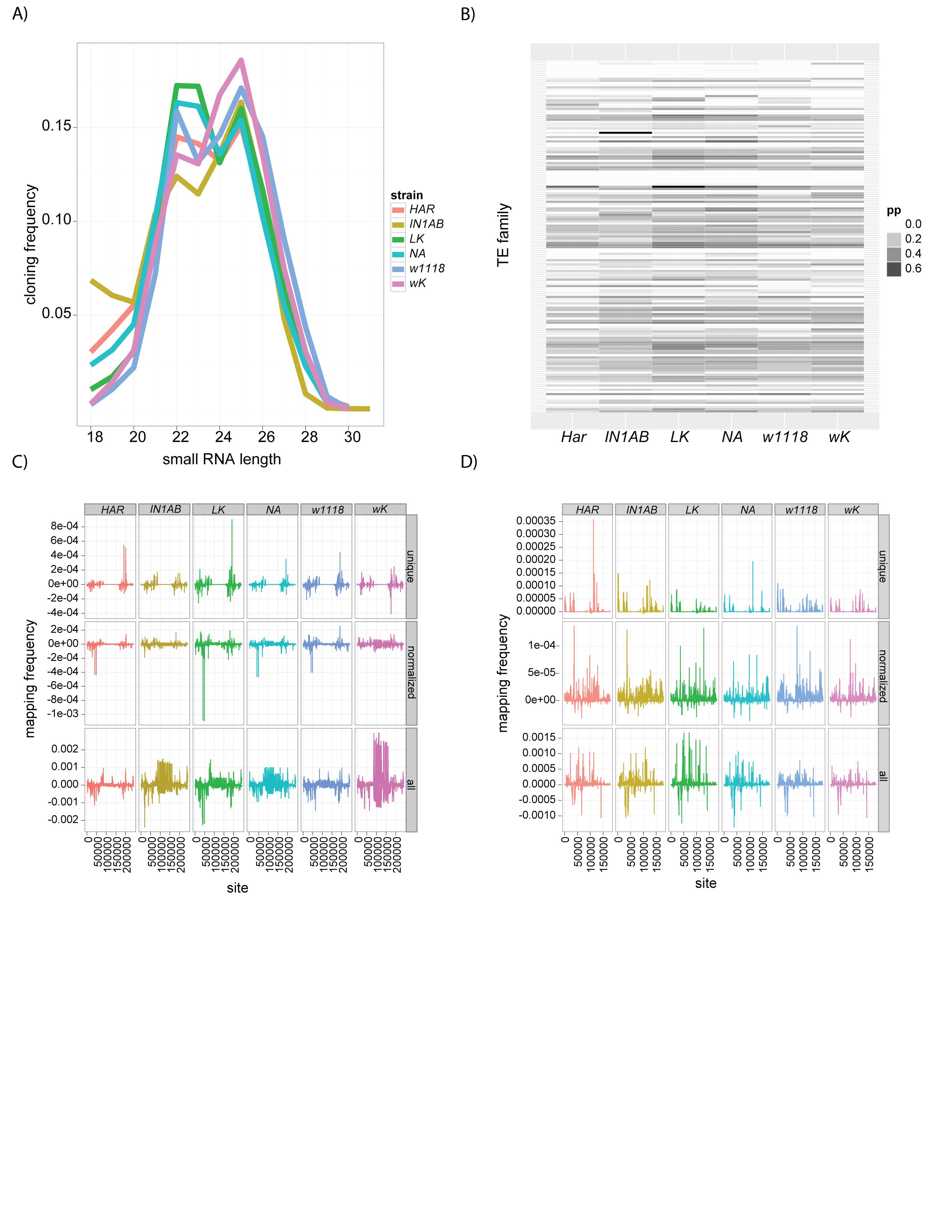

Supplement: Figure S1 — In(1)AB, Hmr2 heterozygotes exhibit a wild-type piRNA pool. (A) The size distribution of cloned small RNAS from In(1)AB, Hmr2/FM6 ovaries is similar to that of other wild-type strains. (B) The ping pong fraction [23] of TE families sampled in In(1)AB, Hmr2/FM6 piRNAs is similar to that of other wild-type strains. (C and D) The frequency of reads mapping to the 42AB and flamenco piRNA clusters is similar between In(1)AB, Hmr2/FM6 and other wild-type strains. Wild-type small RNA libraries are from [23]. (TIF) [file pbio.1001428.s001.tif]

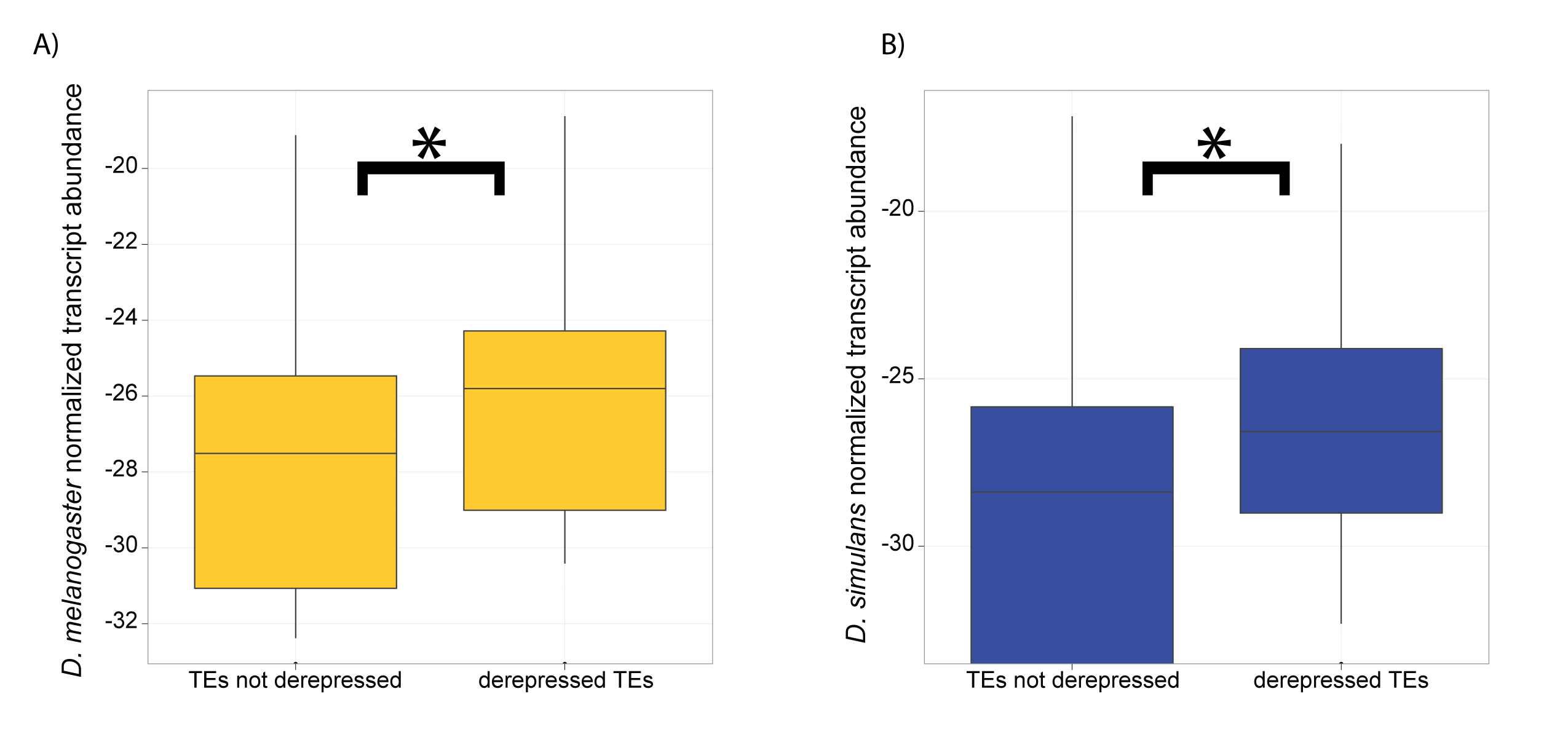

Supplement: Figure S2 — Positive relationship between normalized TE transcript abundance in the ovarian mRNAs of parental pure species and TE derepression in interspecific hybrids. Log2 TE-derived transcript abundance in D. melanogaster (A) and D. simulans (B) ovarian mRNAs is compared for TE families not derepressed and derepressed in interspecific hybrids. TE transcript abundance was normalized by library size and the length of the consensus sequence for each TE family. Derepressed TEs were those whose transcript abundance increased 2-fold or more in interspecific hybrids when compared to their parental pure species, regardless of whether this increase was statistically significant. TE families derepressed in interspecific hybrids showed a higher average TE transcript abundance in parental pure species than those that were not derepressed (* Wilcoxon Rank-Sum p<0.05). These comparisons complement those presented in the main text (Figure 2B–C), where TE families were considered derepressed only if the 2-fold or greater increase in transcript abundance represented a significant difference in expression between the hybrids and their parents (q-value<0.05). The requirement of a statistically significant increase in expression could bias towards TE families with higher transcript abundance becoming derepressed in interspecific hybrids, because these TEs have a higher read count and thus more power to reject the null hypothesis of no difference in expression. However, because differences in TE transcript abundance in parental pure species are robust, even in the absence of a requirement that that the 2-fold or greater increased expression in interspecific hybrids is statistically significant (A–B), we conclude that they are not an artifact of statistical power. (TIF) [file pbio.1001428.s002.tif]

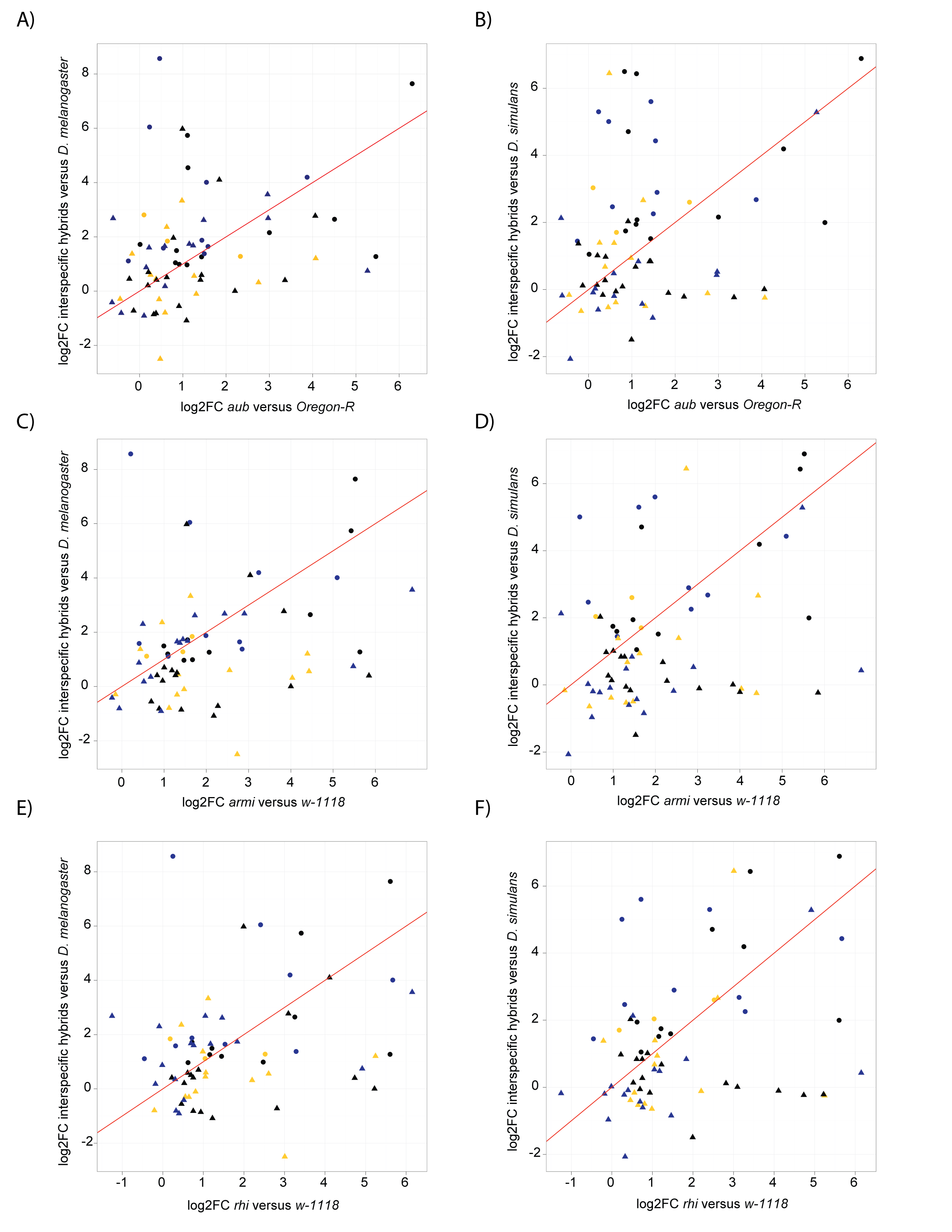

Supplement: Figure S3 — Correlations between TE activity in interspecific hybrids and piRNA pathway mutants. Colors denote TE classes more abundant among D. melanogaster piRNAs (yellow), D. simulans piRNAs (blue), or nondifferentially abundant between the piRNAs of these two species (black), from Figure 1C. Circles denote TE classes inferred as derepressed in interspecific hybrids, whereas triangles denote TEs not derepressed in interspecific hybrids (as in Figure 1A). Red line denotes equivalent changes in expression in hybrids relative to parental pure species when compared to piRNA mutants [44],[45] relative to wild-type controls (w1118). (A) Interspecific hybrid/D. melanogaster versus aub/Oregon-R (Pearson's r = 0.30, p = 0.01). (B) Interspecific hybrid/D. simulans versus aub/Oregon-R (Pearson's r = 0.27, p = 0.03). (C) Interspecific hybrid/D. melanogaster versus armi/w1118 (Pearson's r = 0.29, p = 0.02). (D) Interspecific hybrid/D. simulans versus armi/w1118 (Pearson's r = 0.35, p = 0.004). (E) Interspecific hybrid/D. melanogaster versus rhi/w1118 (Pearson's r = 0.29, p = 0.02). (F) Interspecific hybrid/D. simulans versus rhi/w1118 (Pearson's r = 0.35, p = 0.004). After accounting for the correlations among different piRNA mutants, only the correlation between hybrids/D. simulans and ago3/wild-type D. melanogaster remains significant (Pearson's r = 0.4, p = 0.0054, Table S4). (TIF) [file pbio.1001428.s003.tif]

A)

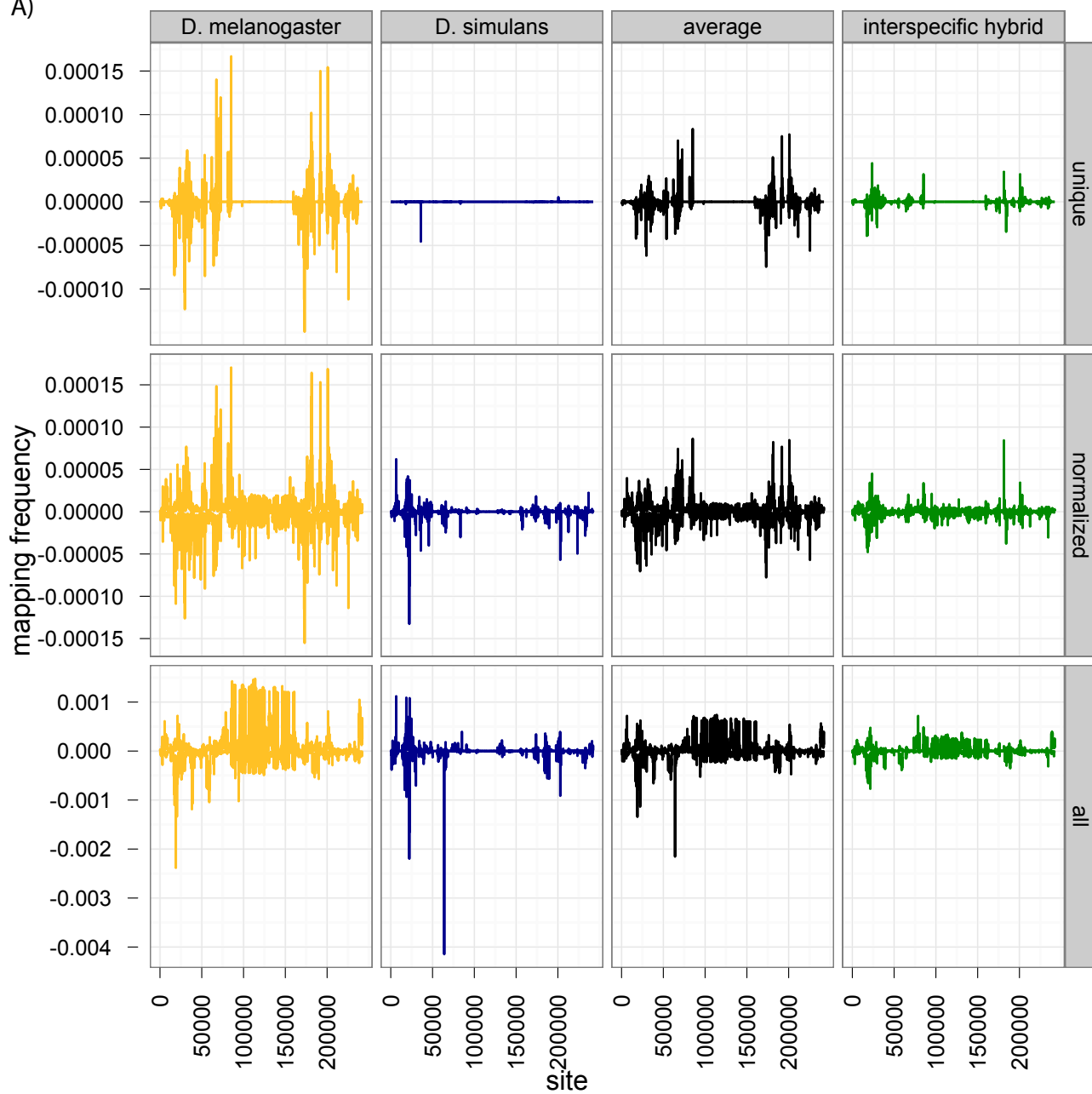

B)

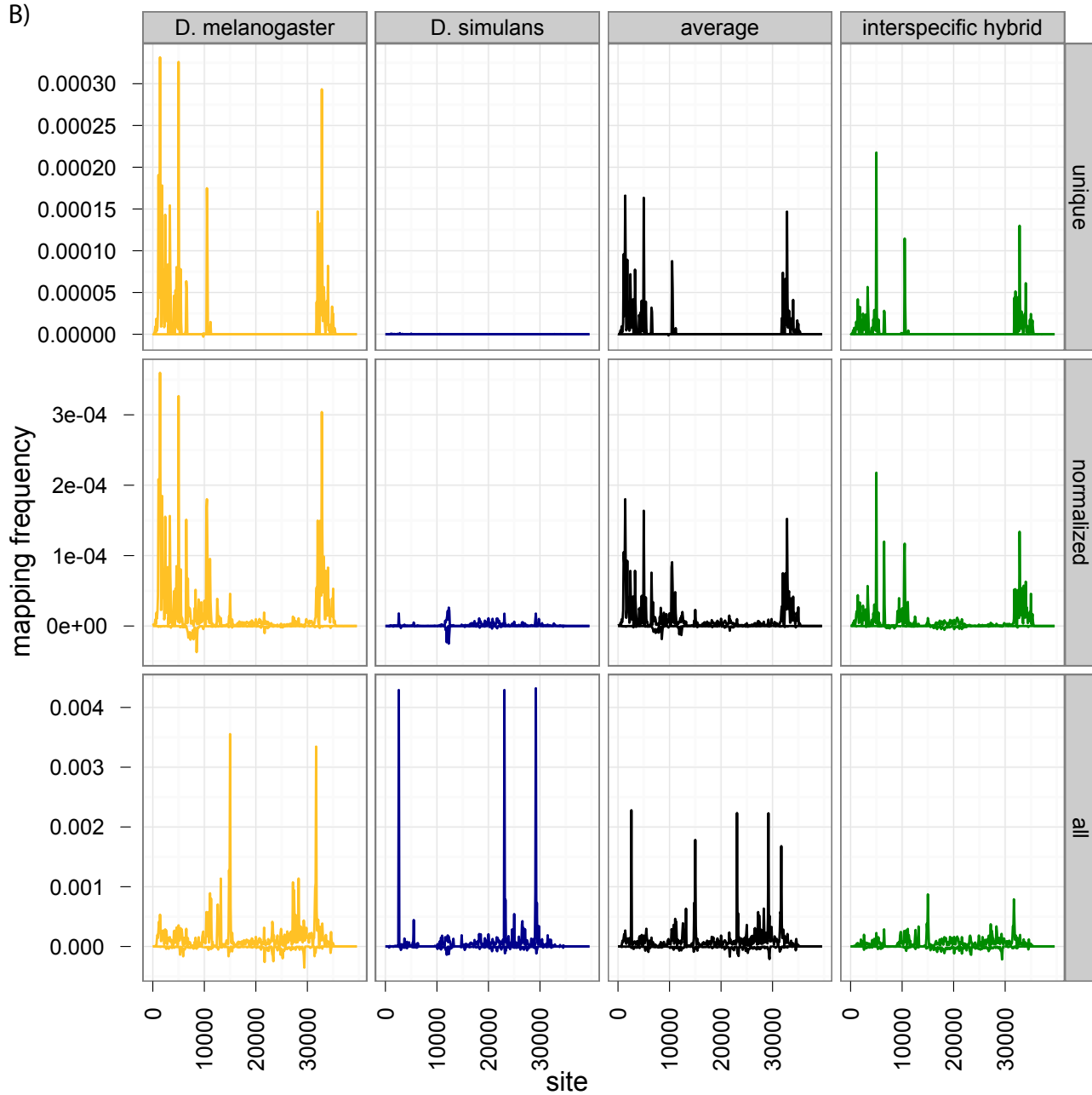

B)

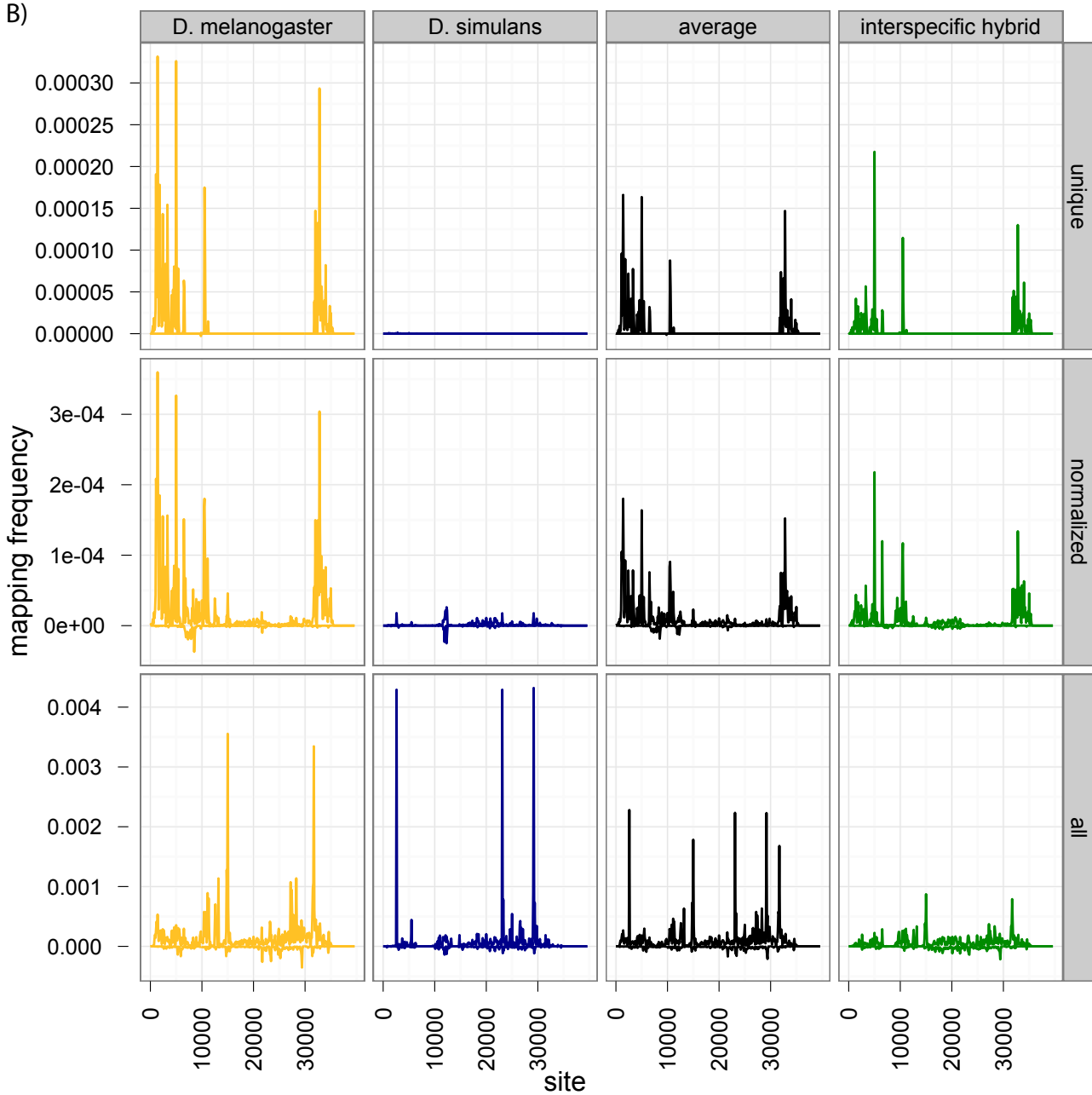

B)

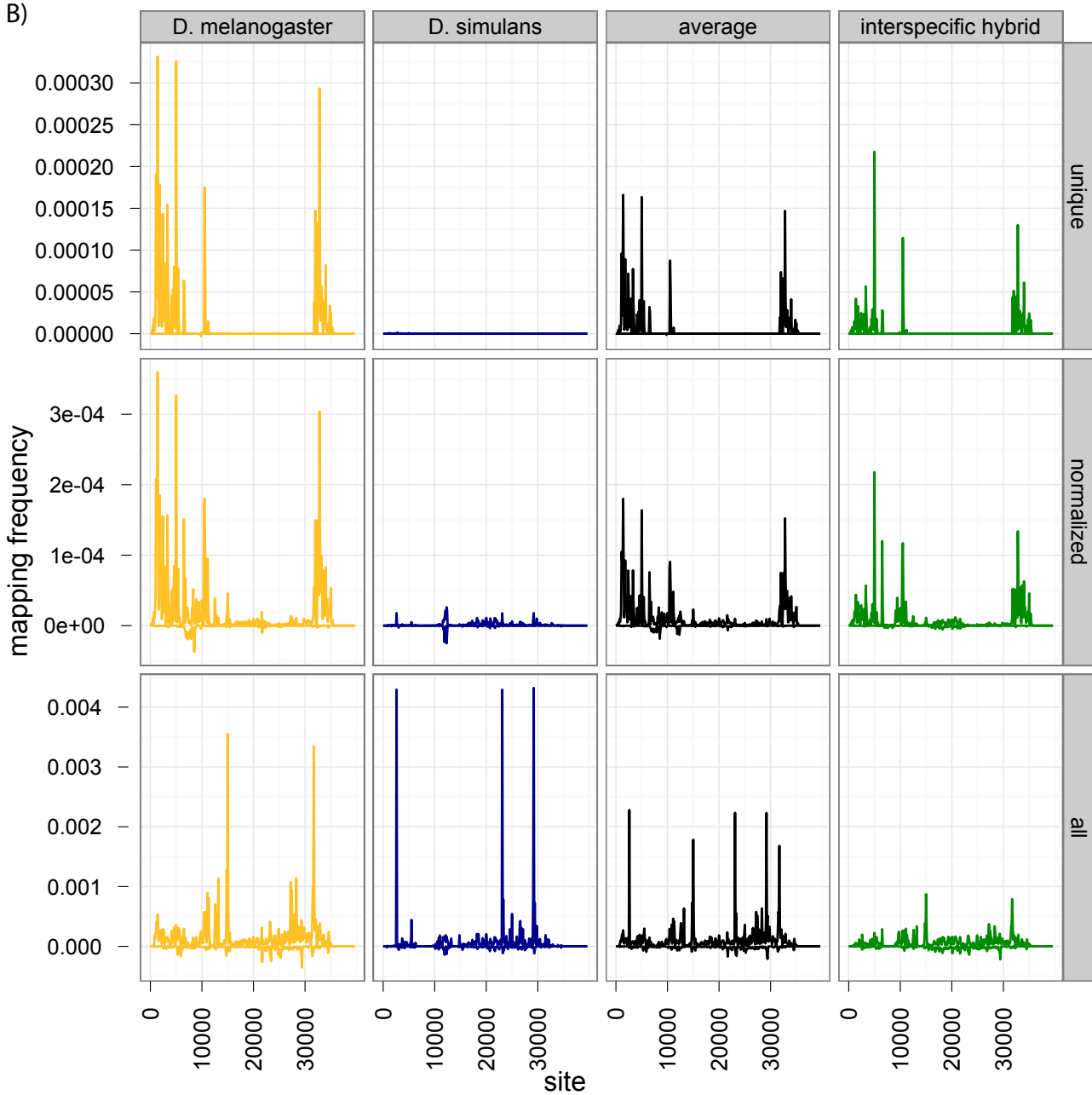

C)

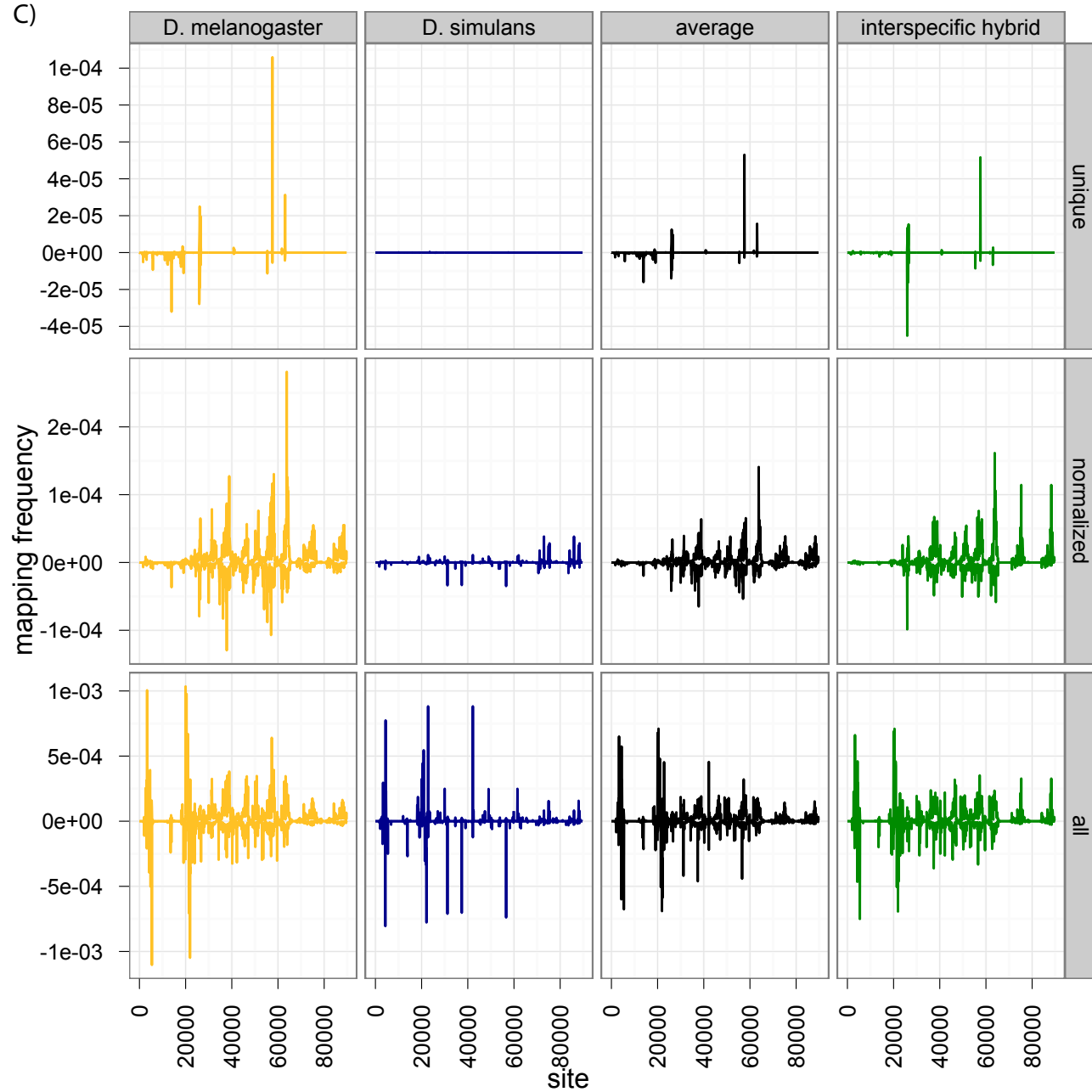

D)

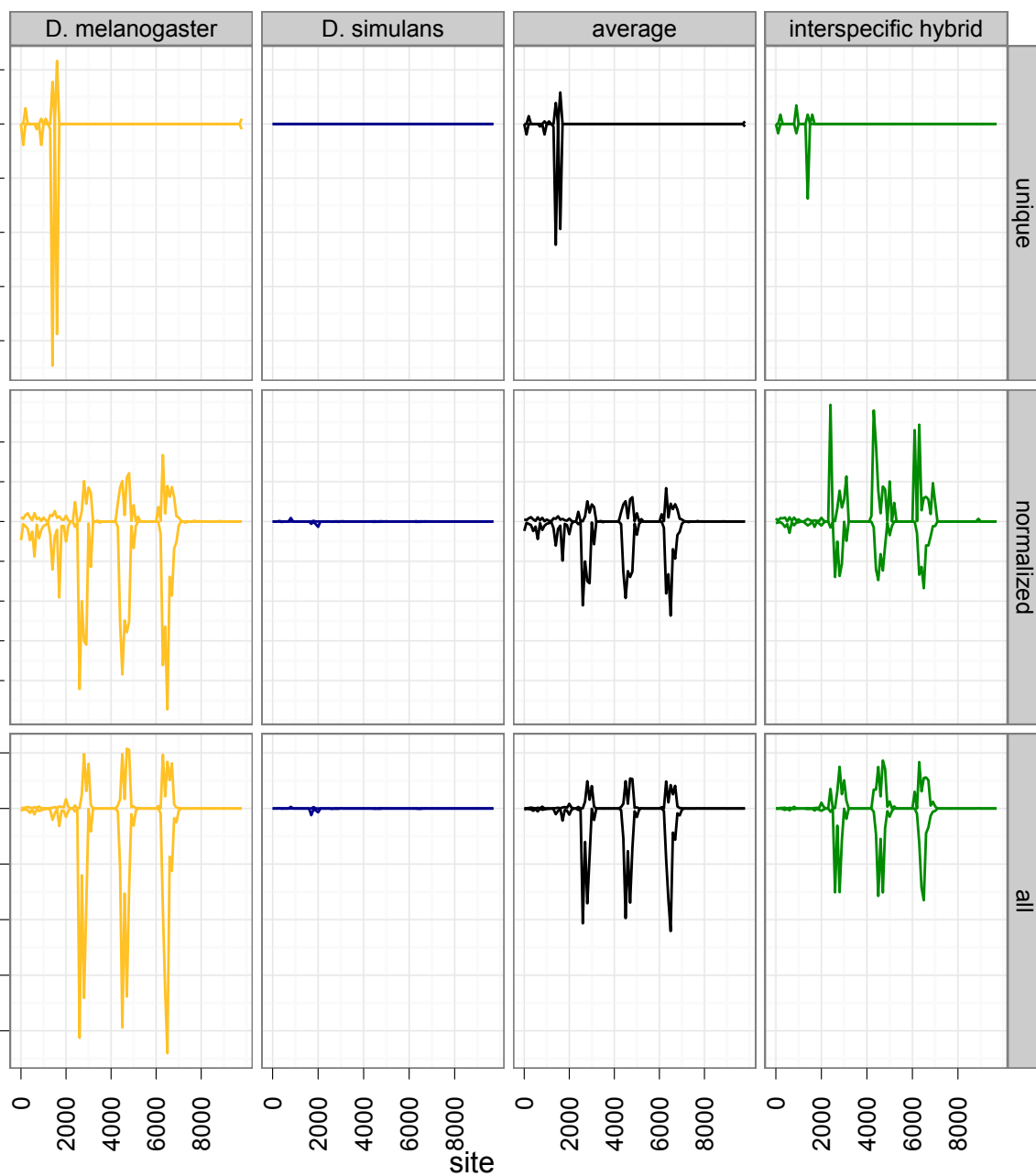

E)

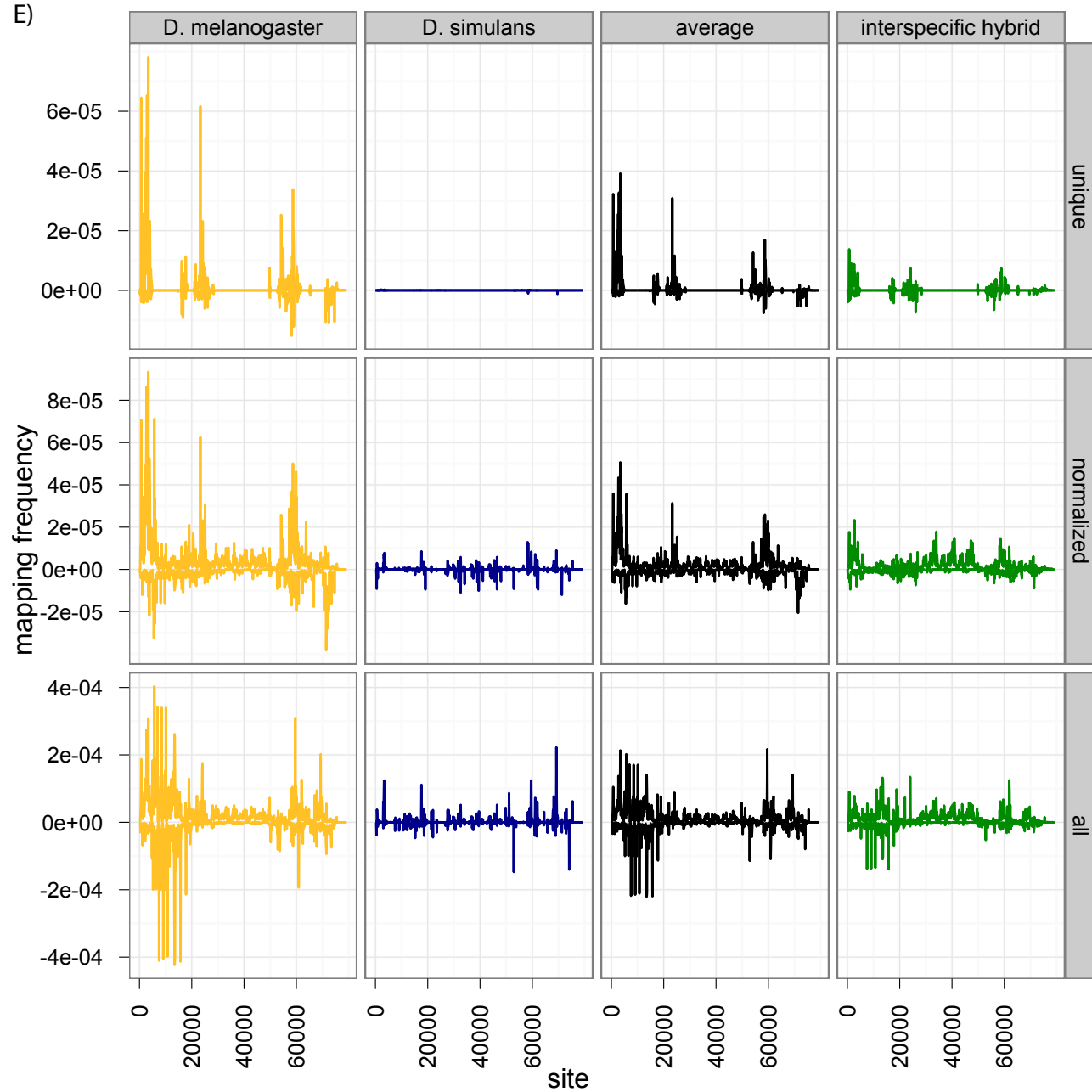

E)

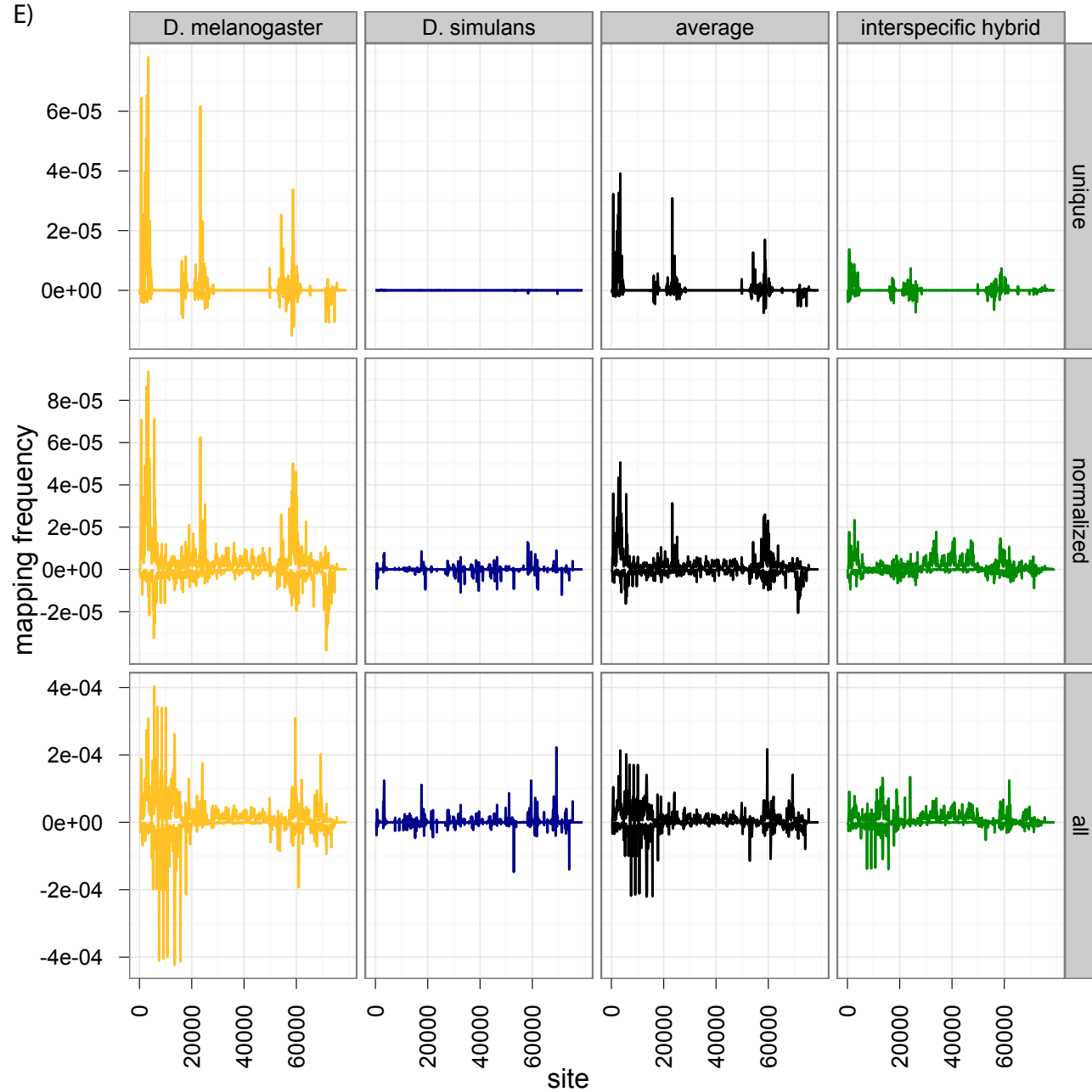

F)

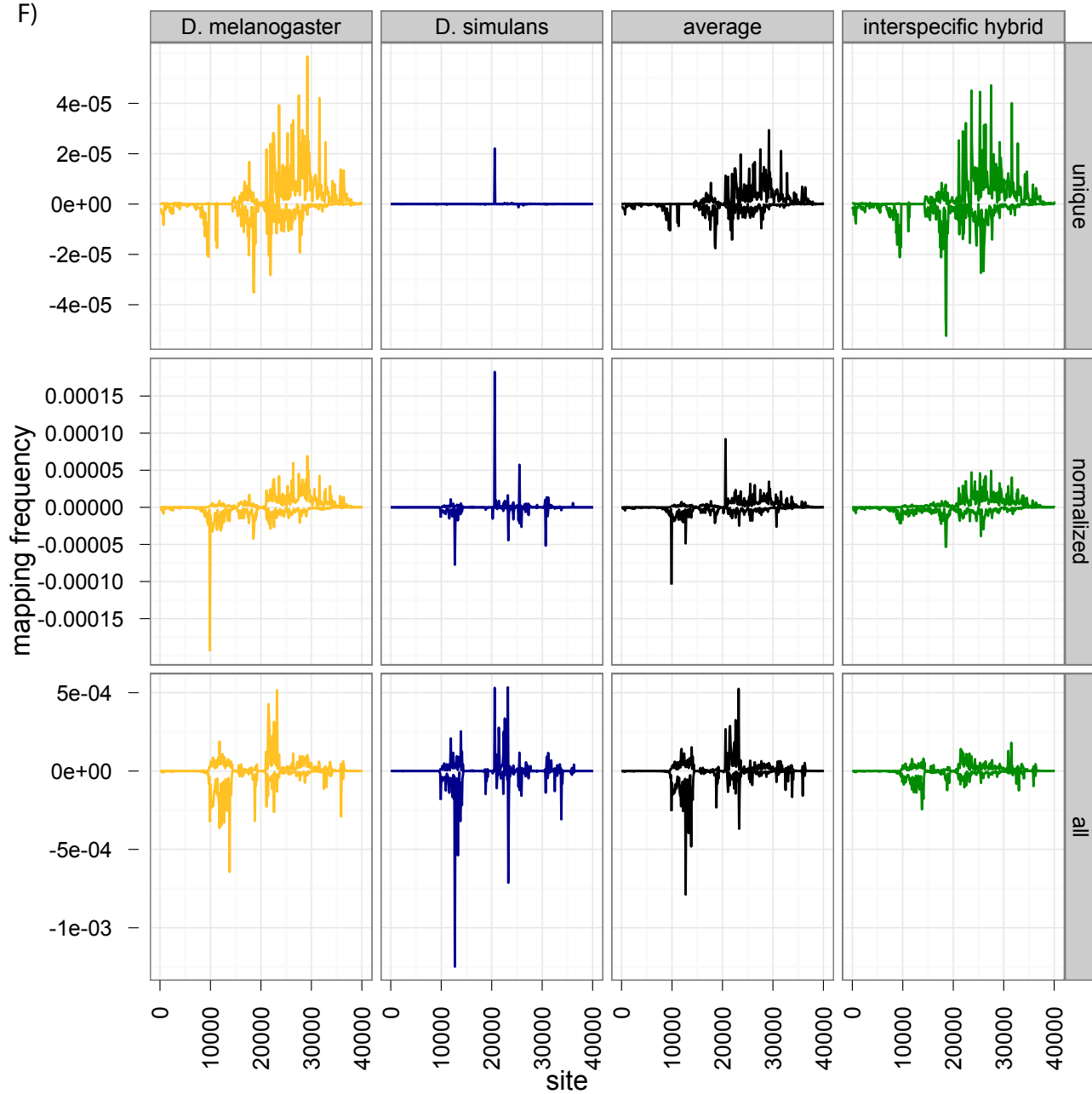

G)

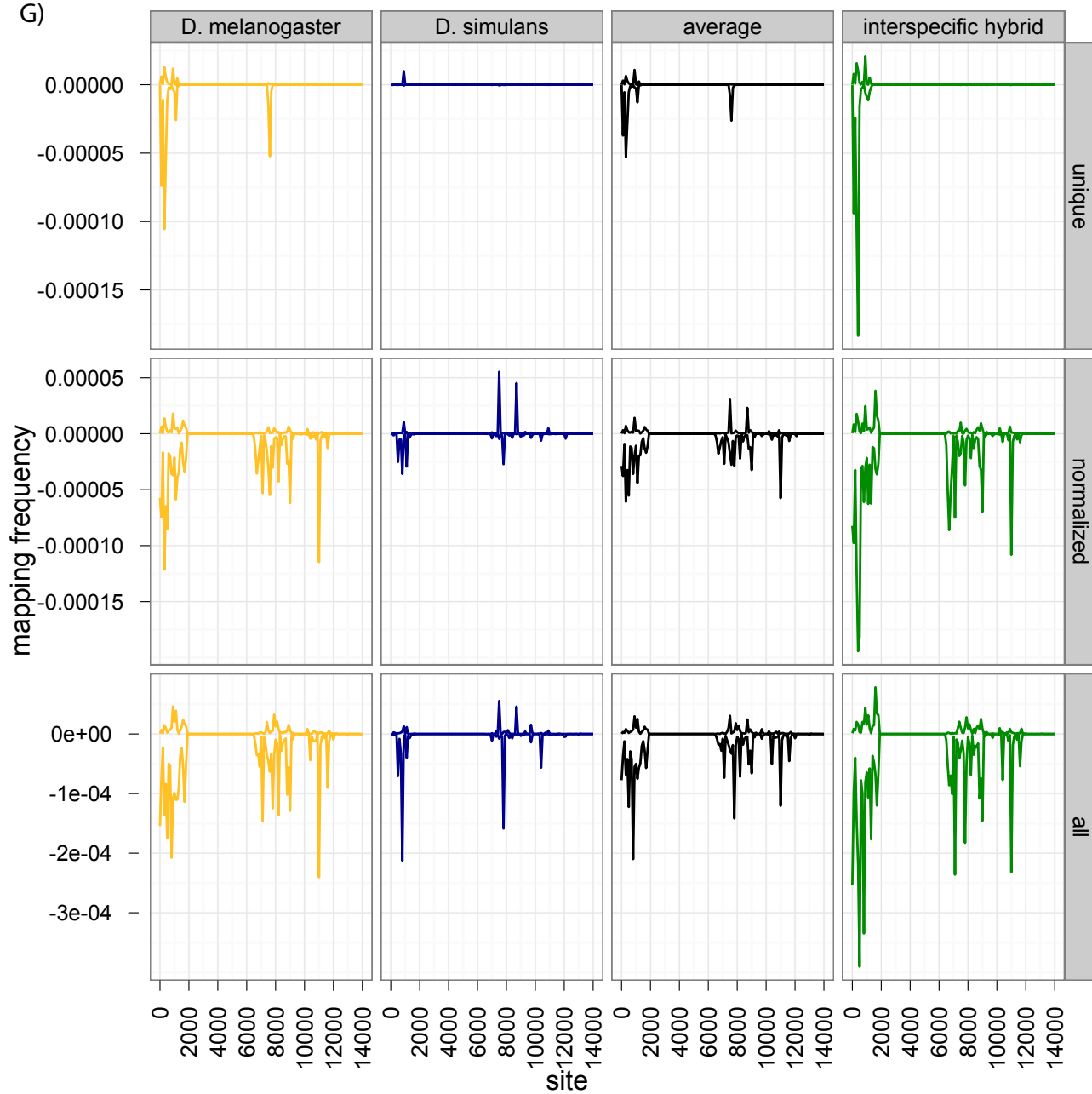

H)

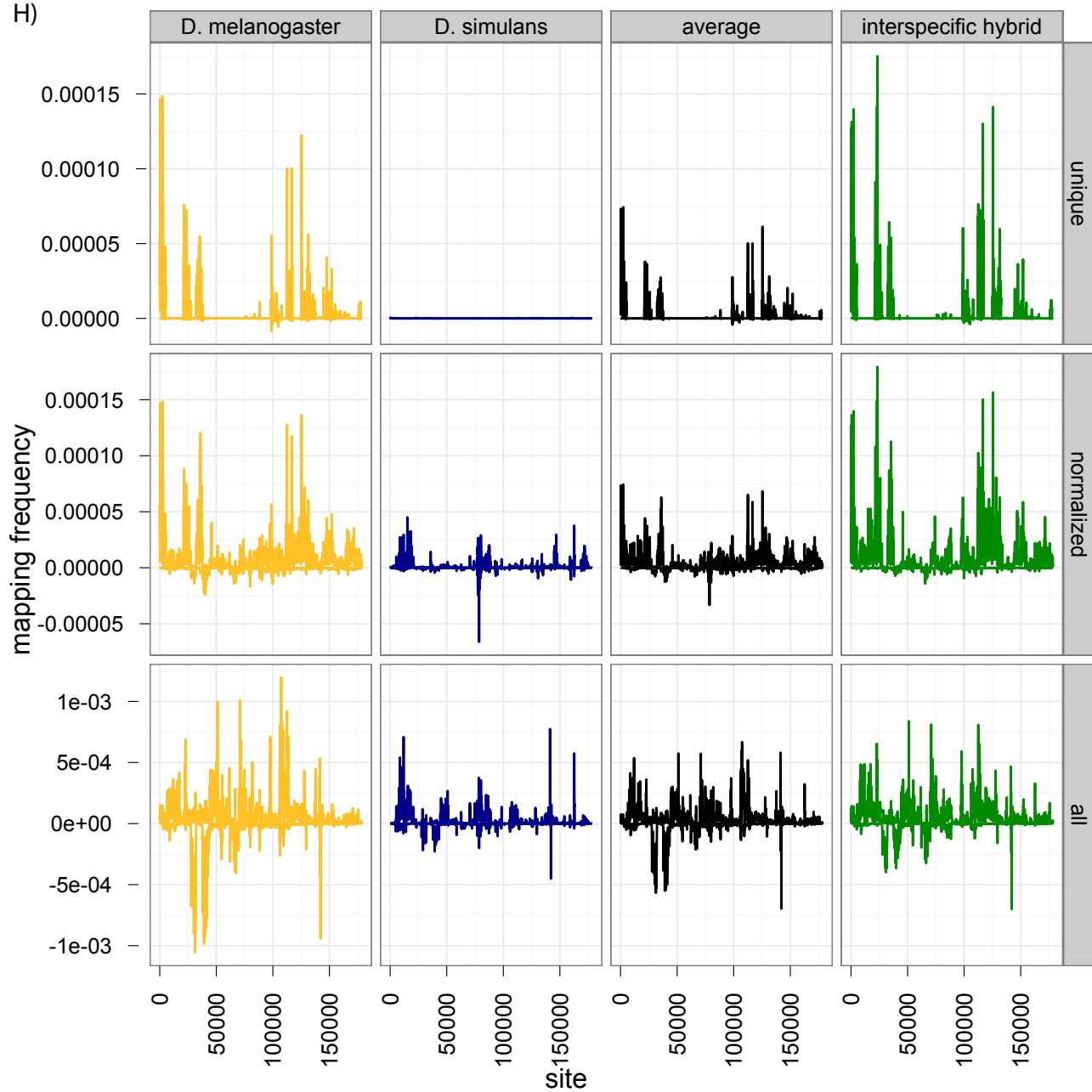

1)

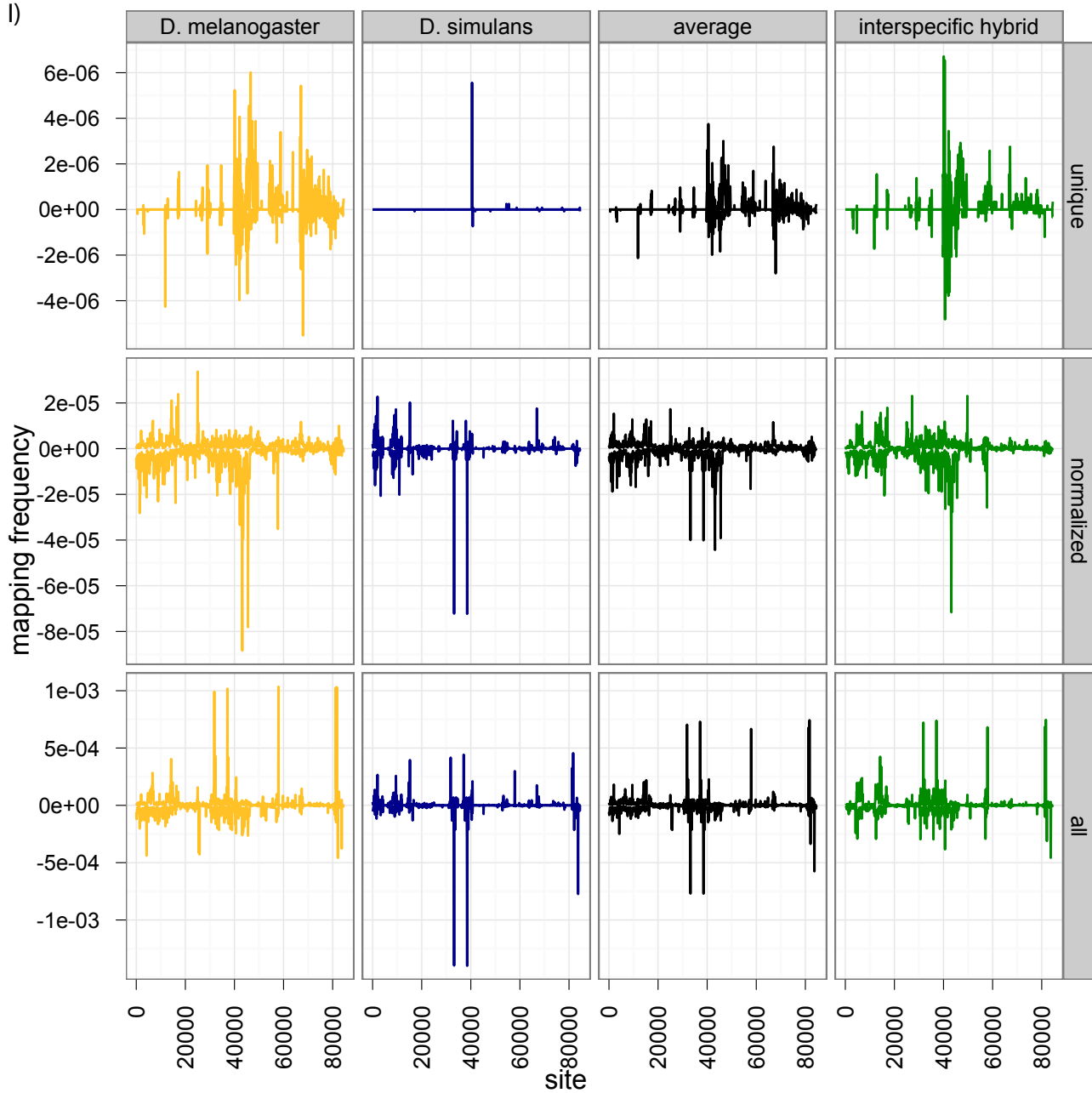

J)

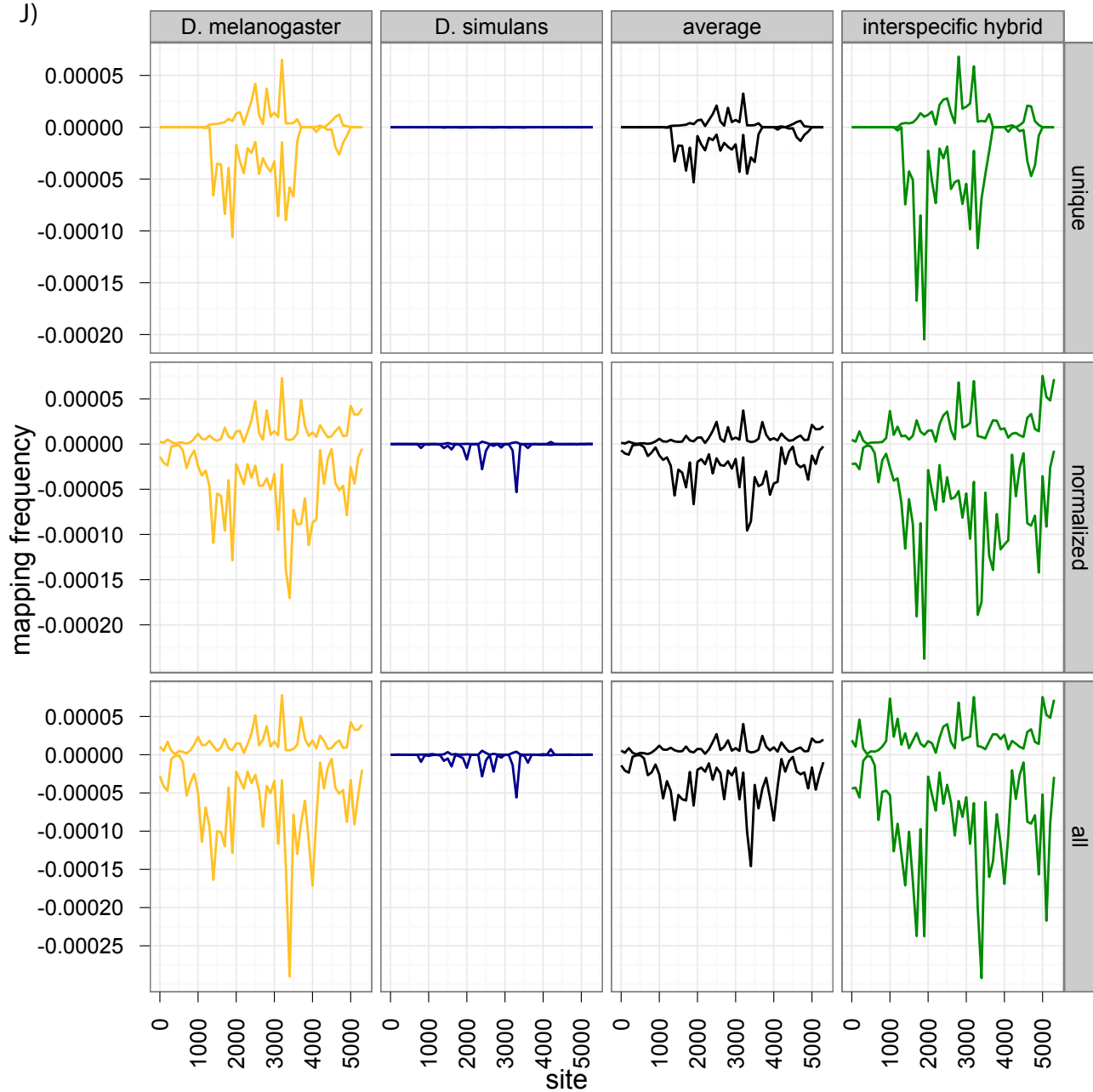

K)

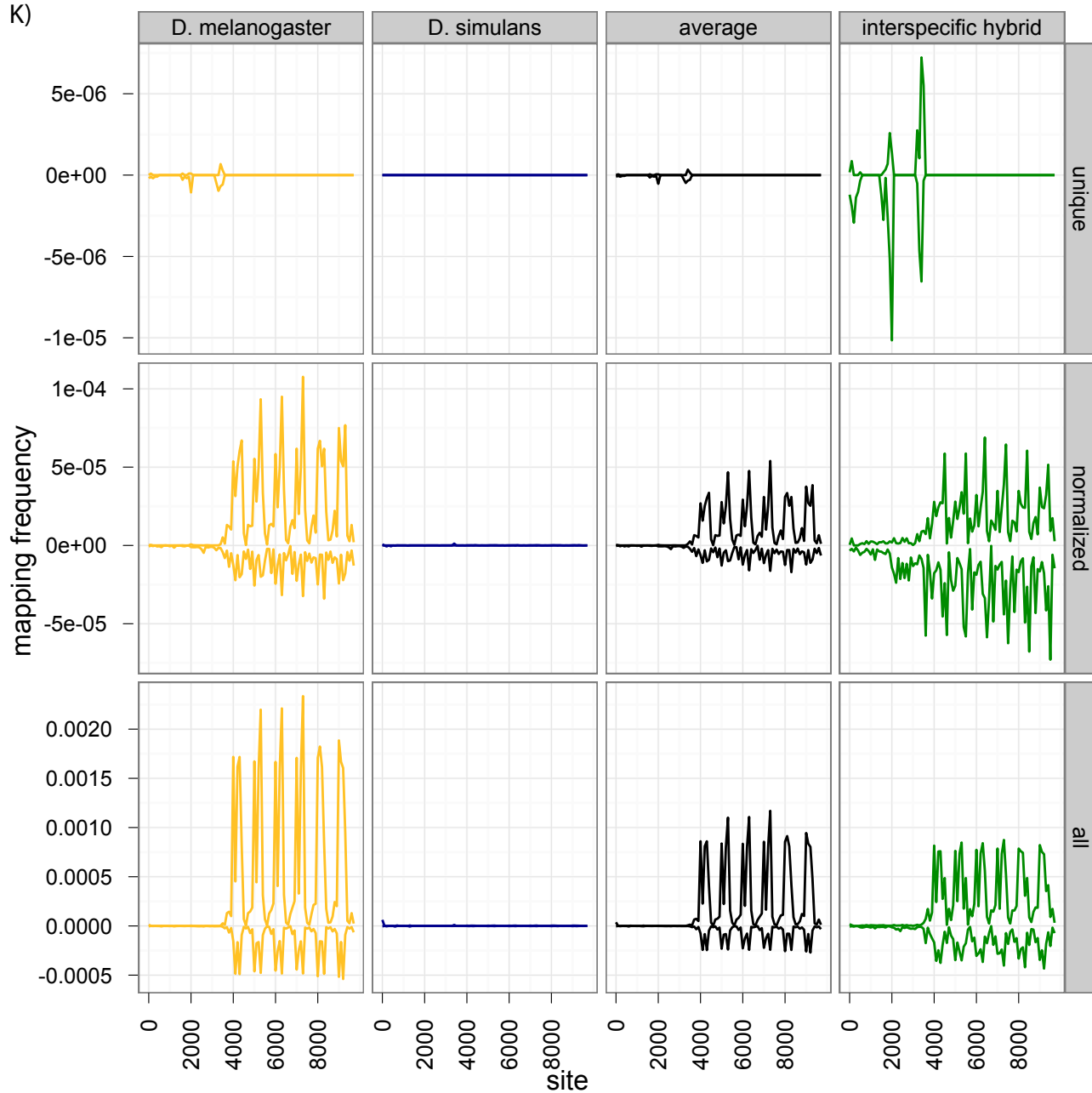

L)

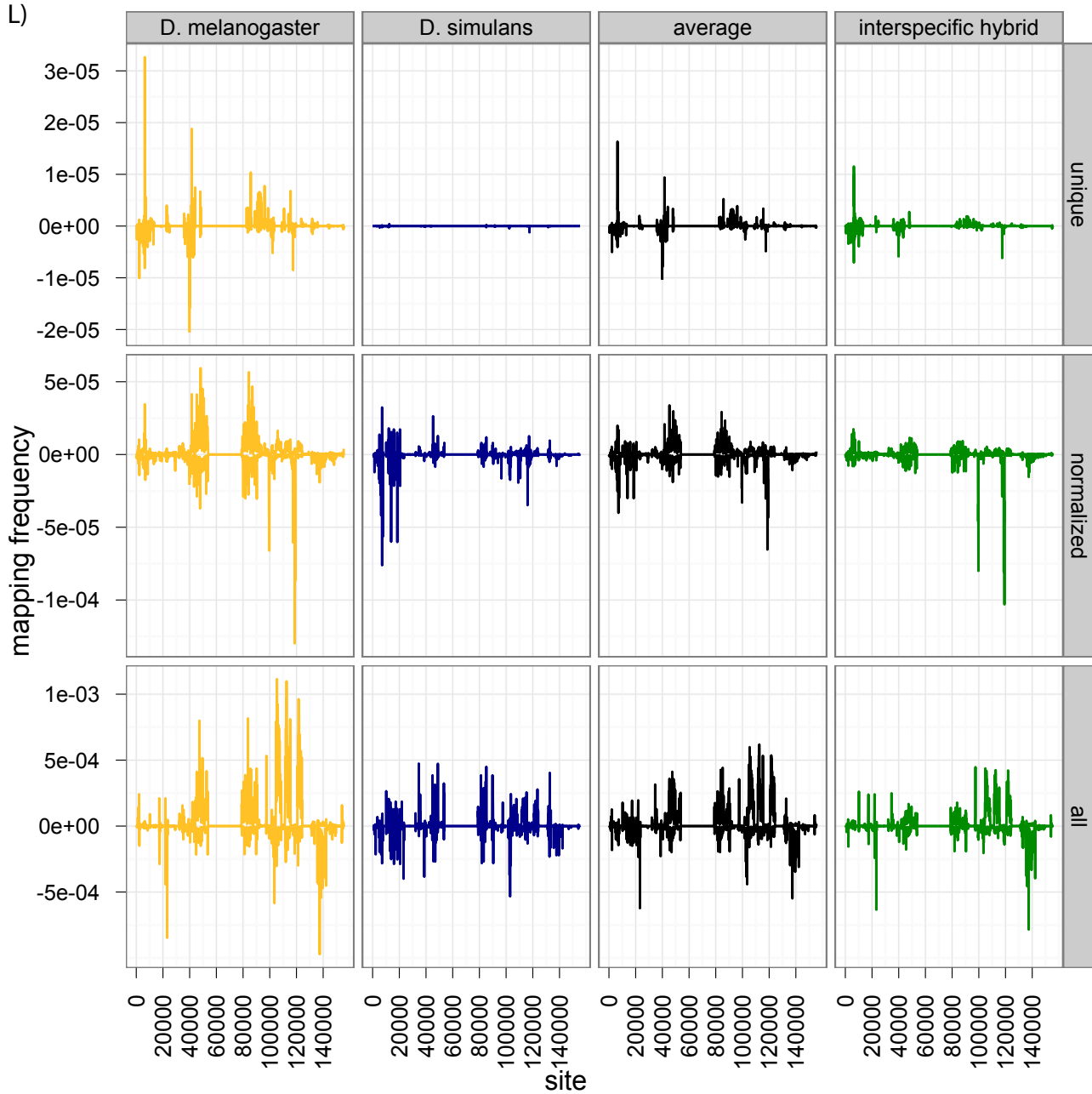

M)

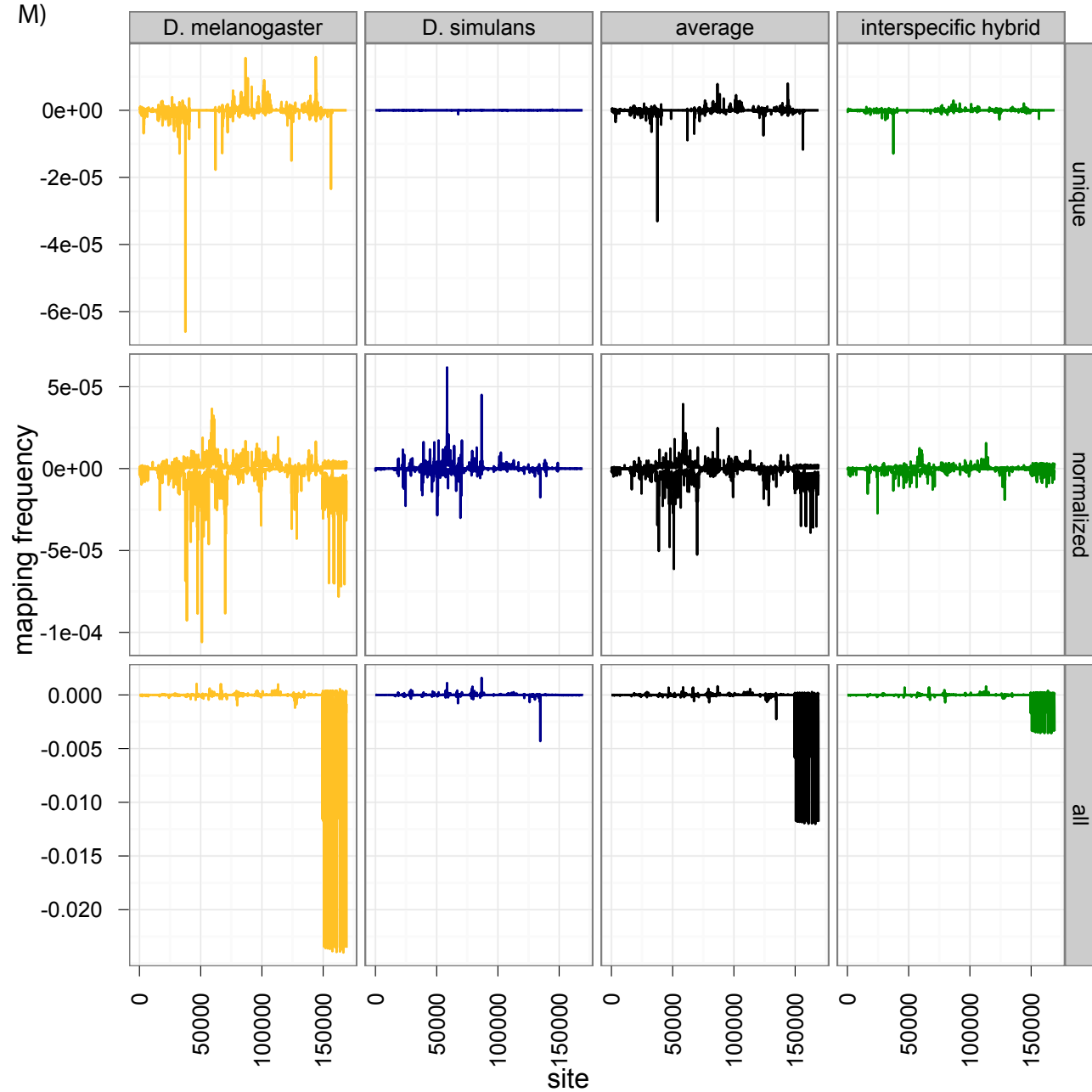

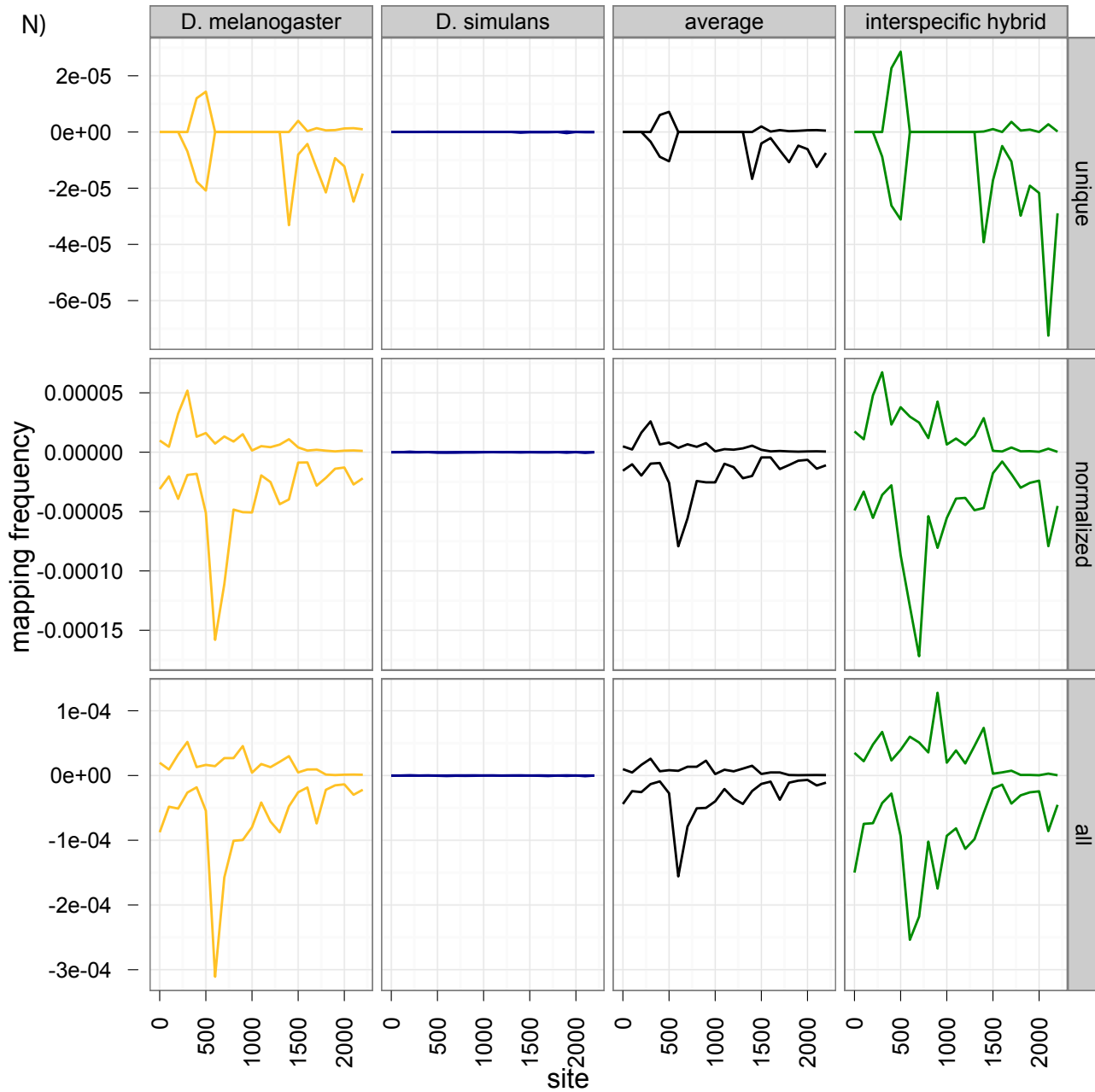

O)

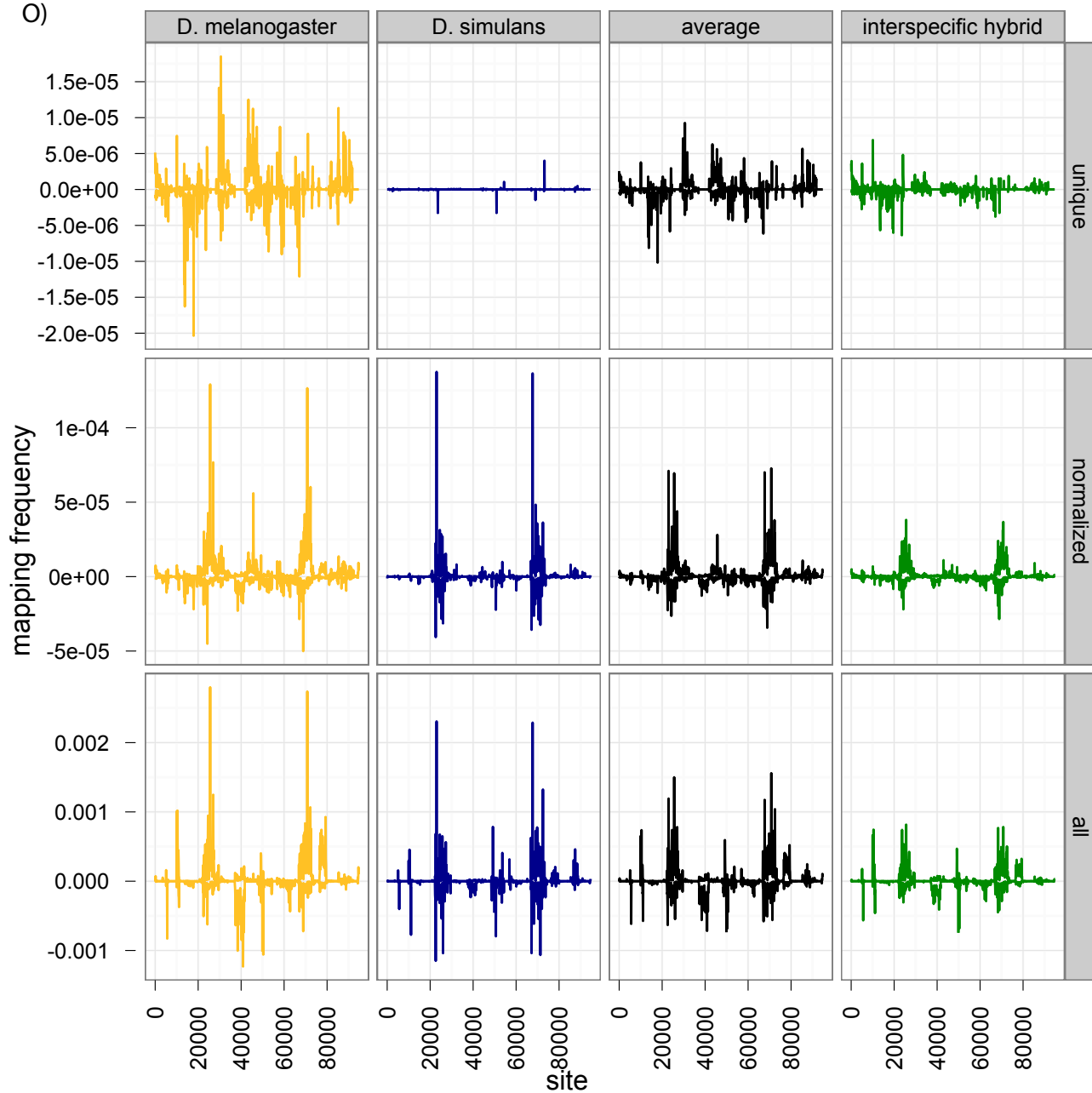

Supplement: Figure S4 — Proportions of piRNA reads mapping to 15 heterochromatic piRNA clusters described in Brennecke et al. [17]. Frequency of D. melanogaster (yellow), D. simulans (blue), and interspecific hybrid (green) piRNAs mapping with zero mismatches to the piRNA cluster are shown. An additive interspecific average also is shown (black) as a prediction for hybrid read mapping. Top, reads mapping uniquely to the cluster. Middle, all reads mapping to the cluster, normalized by the number of other genomic mapping locations with zero mismatches. Bottom, all reads mapping to the cluster. (PDF) [file pbio.1001428.s004.pdf]

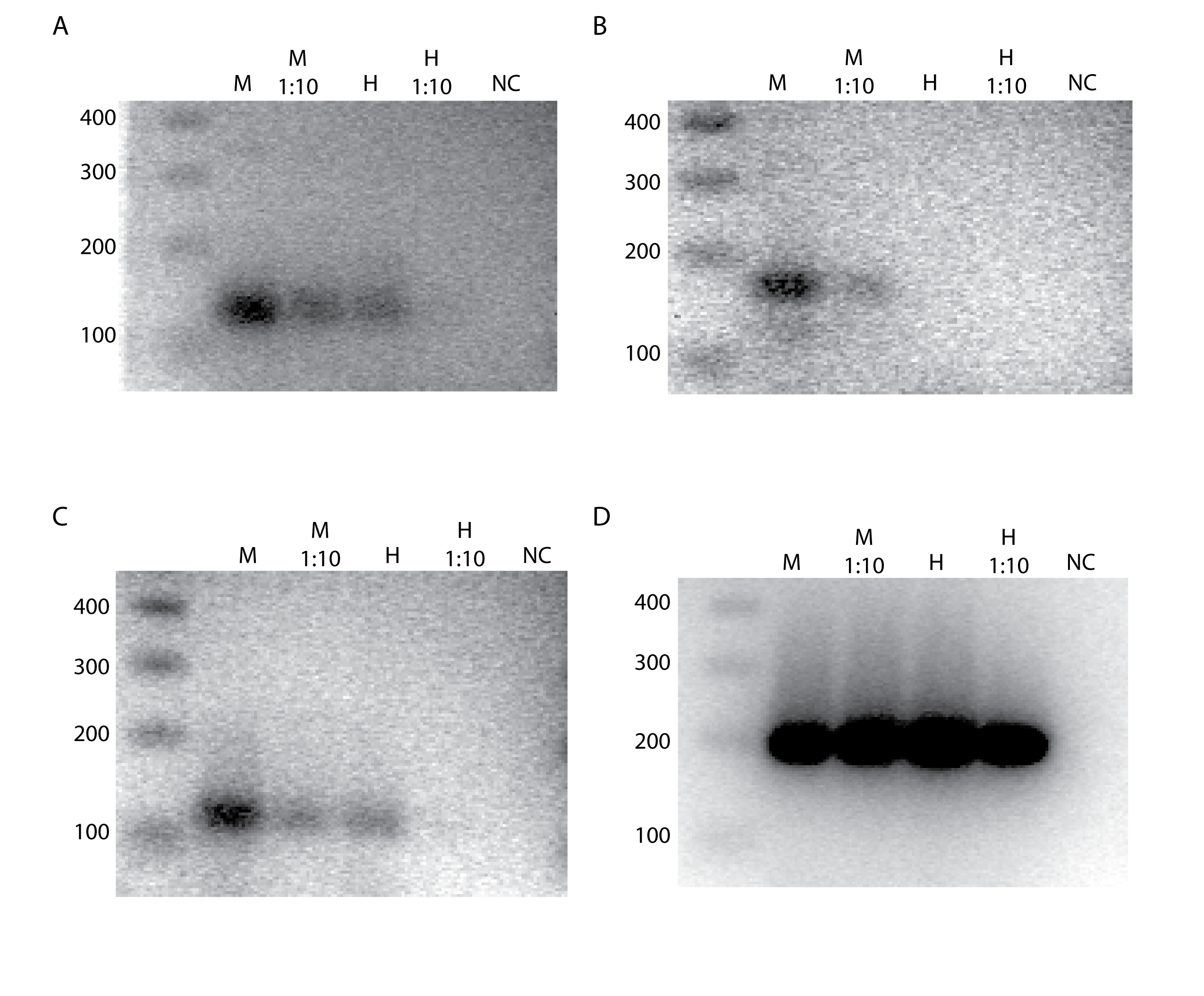

Supplement: Figure S5 — Semi-quantitative RT-PCR of piRNA precursor transcripts. Precursor transcript was amplified in a 20 µL PCR using 5 µL of 500 ng/µL D. melanogaster cDNA (M), 500 ng/µL interspecific hybrid cDNA (H), 50 ng/µL D. melanogaster cDNA (M 1∶10), 50 ng/µL interspecific hybrid cDNA (H 1∶10), or negative control (NC) using (A) Cluster5A, (B) Cluster5B, (C) FlamB, and (D) Rpl32 primer pairs. (TIF) [file pbio.1001428.s005.tif]

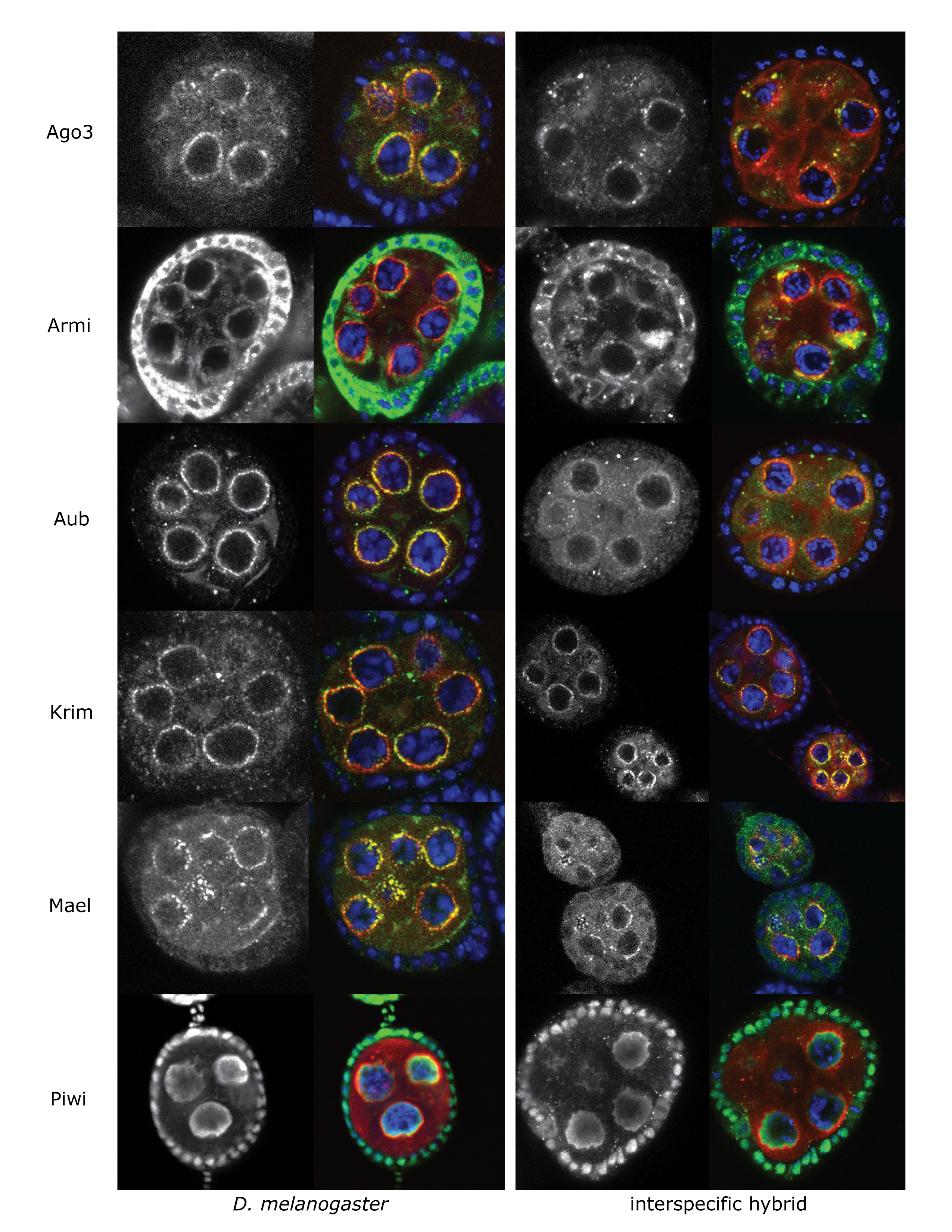

Supplement: Figure S6 — Nuage localization of piRNA proteins in interspecific hybrids. Interspecific hybrids were compared with their D. melanogaster mothers for the localization of 5 nuage components (grey scale). Merged images are colocalization between each protein examined (green) and Vasa protein (red), an additional nuage component. Scale bar: 10 µm. (TIF) [file pbio.1001428.s006.tif]

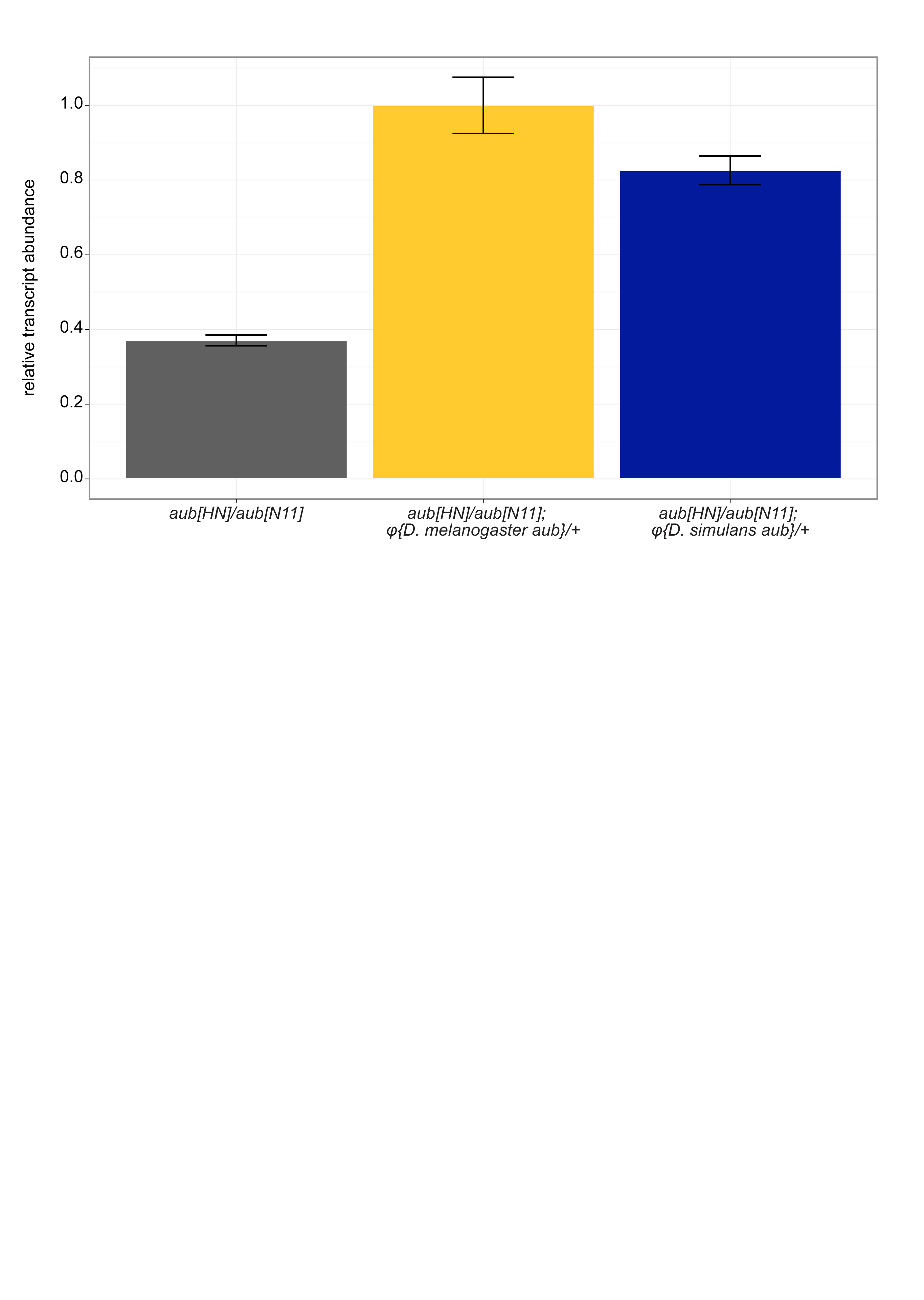

Supplement: Figure S7 — Quantitative RT-PCR of aubergine in mutant and transgenic backgrounds. TE transcript abundance was determined relative to rpl32 and is scaled to the transcript abundance in aub[HN]/aub[N11];φ{D. melanogaster aubergine}/+. (TIF) [file pbio.1001428.s007.tif]

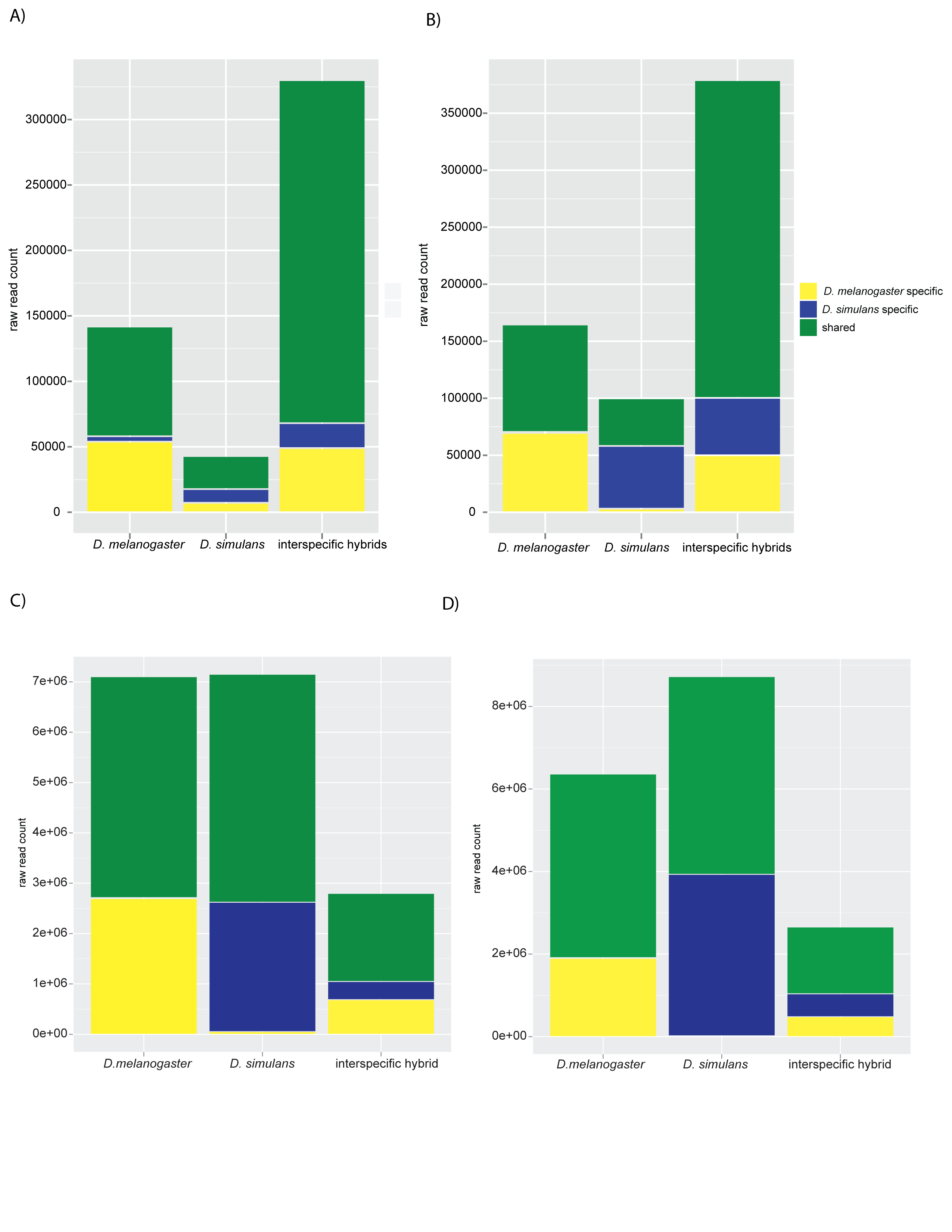

Supplement: Figure S8 — Identification of TE-derived mRNAs and piRNAs. Yellow indicates TE-derived reads unique to the D. melanogaster genome, blue indicates TE-derived reads unique to the D. simulans genome, and green indicates TE-derived reads that are found in either genome. (A) mRNAs mapped to a database of consensus TEs. (B) mRNAs mapped to all annotated TE insertions in both the D. melanogaster and D. simulans genomes. (C) piRNAs mapped to a database of consensus TEs. (D) piRNAs mapped to a database of all annotated TE insertions in both the D. melanogaster and D. simulans genomes. More piRNA and mRNA reads that are unique to the D. simulans genome are identified as TEs when the database of all annotated TEs is used. (TIF) [file pbio.1001428.s008.tif]

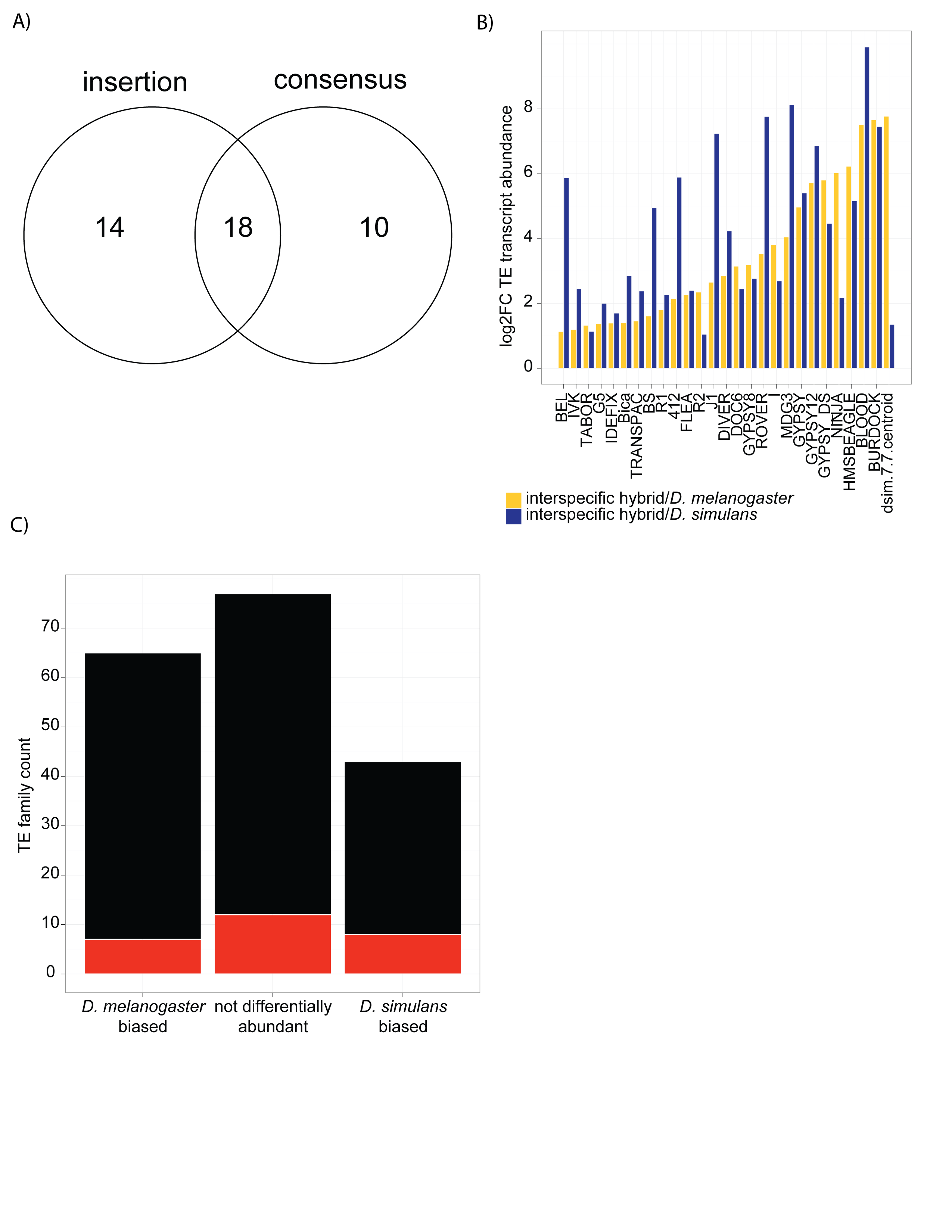

Supplement: Figure S9 — Identifying TE-derived reads by mapping to a consensus sequence. (A) Venn diagram of overlap between TE classes identified as derepressed in interspecific hybrids using the insertion mapping and consensus mapping approaches. (B) TE classes identified as derepressed using the consensus mapping approach. (C) Relationship between interspecific divergence in piRNA abundance for individual TE classes and derepression of those TE classes, when TE-derived piRNAs are identified by mapping to a consensus sequence. TE classes were categorized as D. melanogaster biased, D. simulans biased, or nondifferentially abundant, based on their relative abundance in D. melanogaster and D. simulans piRNAs. The proportion of TE classes in each of these categories that are derepressed in interspecific hybrids is indicated by the area shaded in red. (TIF) [file pbio.1001428.s009.tif]

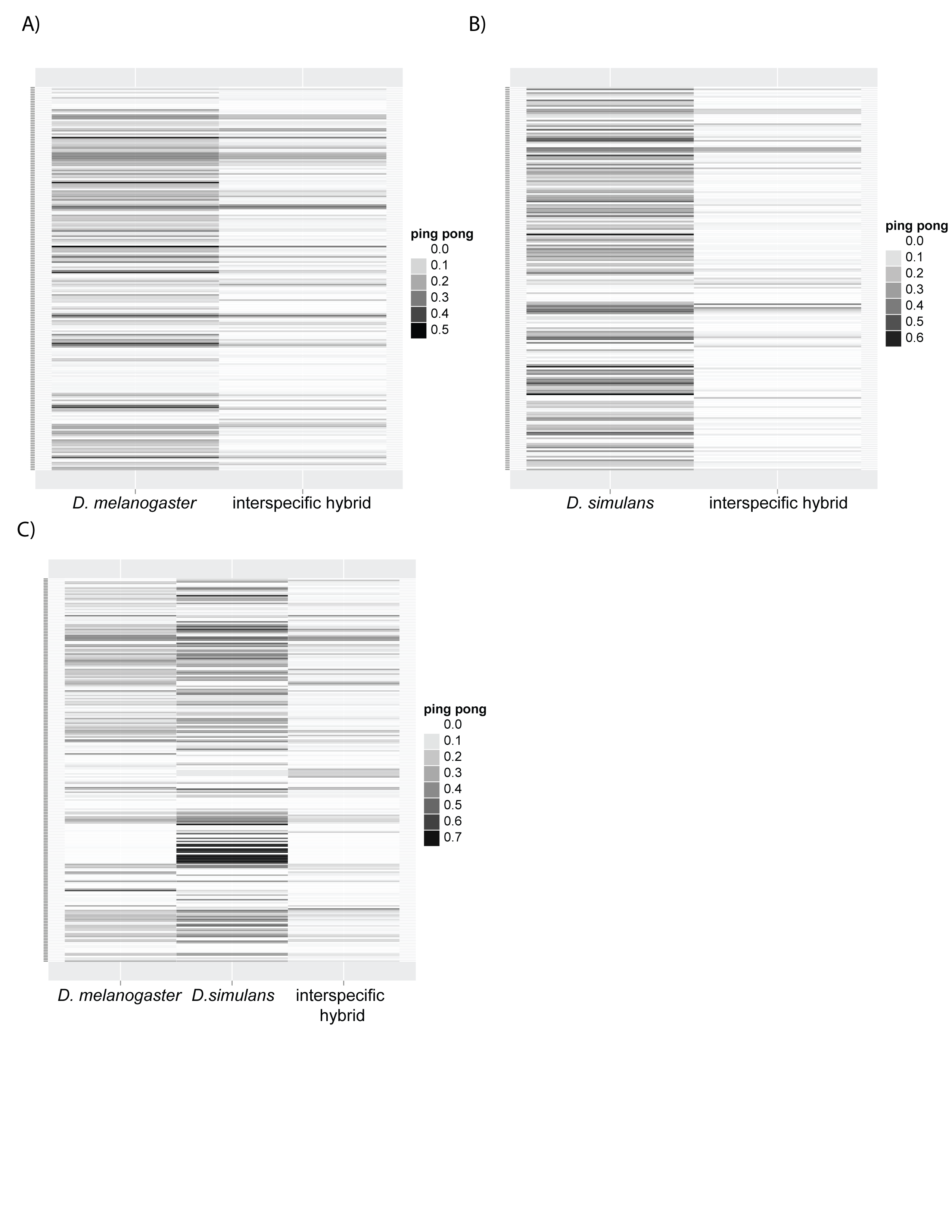

Supplement: Figure S10 — Ping pong fraction [23] calculated for piRNA reads mapping to the D. melanogaster genome only (A), to the D. simulans genome only (B), and to both genomes (C). Interspecific hybrids are compared to their parental pure species, D. melanogaster and D. simulans. (TIF) [file pbio.1001428.s010.tif]

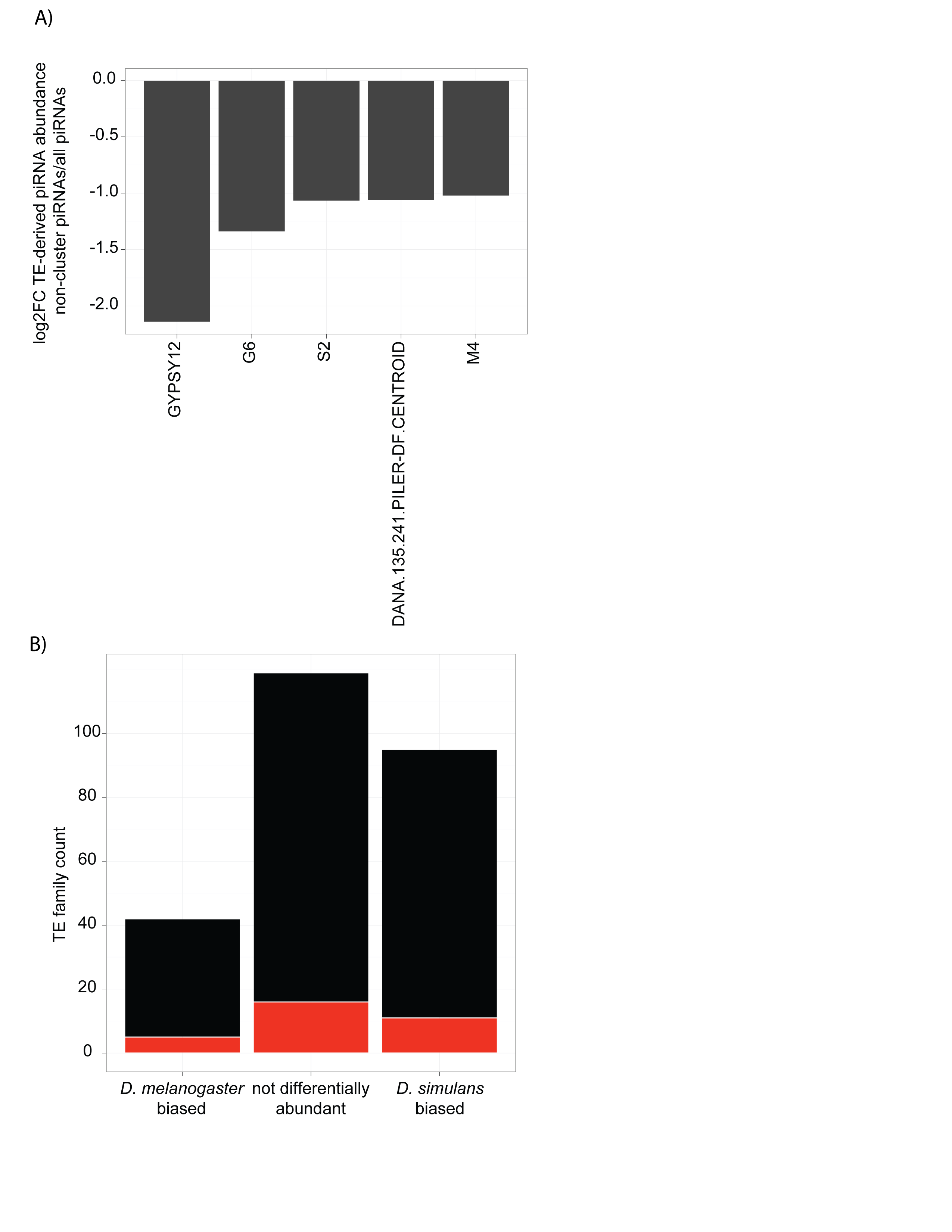

Supplement: Figure S11 — Data reanalysis excluding cluster-derived piRNAs. (A) Only five TE classes exhibit significant change in abundance within D. melanogaster piRNAs when piRNAs uniquely mapping to heterochromatic clusters are excluded from the analysis. (B) Relationship between interspecific divergence in piRNA abundance for individual TE classes and derepression of those TE classes. TE classes were categorized as D. melanogaster biased, D. simulans biased, or nondifferentially abundant, based on their relative abundance in D. melanogaster and D. simulans piRNAs. The proportion of TE classes from each of these categories that are derepressed in interspecific hybrids is indicated by the area shaded in red. piRNAs uniquely mapping to D. melanogaster piRNA clusters were excluded from each sequencing library before analysis. (TIF) [file pbio.1001428.s011.tif]
