# Supplementary material for: Drosophila Interspecific Hybrids Phenocopy piRNA-Pathway Mutants
Source: PLoS Biol. 2012 Nov 20;10(11):e1001428. doi: 10.1371/journal.pbio.1001428 (PMC3506263; doi:10.1371/journal.pbio.1001428)
Supplement: Text S1 — Supplementary experimental materials and discussion. (DOCX) [file pbio.1001428.s022.docx]

**TEXT S1 CONTENTS**

**Page**

**Supplementary Experimental Procedures** 2

Quality control of Illumina libraries 2

Identification of TE classes and differential expression of TE-derived piRNA and mRNA sequences 2

piRNA cluster transcription 5

Calculation of the ping-pong signature 5

Coding sequence analysis 6

**Supplementary Discussion A: Differences among intraspecific and interspecific hybrid dysgenesis models**

7

**Supplementary Discussion B: Differences in TE annotation and genome assembly between *D. melanogaster* and *D. simulans* and their potential impact on our study.**

Mapping reads to Transposable Elements 10

Antisense fraction and ping-pong fraction 12

piRNA cluster activity 13

**Supplemental References** 14

**Supplementary Experimental Procedures**

Quality Control of Illumina Libraries

Linker sequence was removed from Illumina sequence reads using a custom Perl script. For small RNA libraries, the distance from the start of the read to the start of the 3’ linker sequence indicated the length of the cloned RNA, and reads 23 nt or longer were classified as putative piRNAs. Reads lacking 3’ linker sequence were not analyzed, as their length could not be accurately inferred. Reads were mapped to the *D. melanogaster* genome (release 5) and the *D. simulans* genome (caf1 release/release 1) using Bowtie [1], and all reads that did not align perfectly (0 mismatches) to either genome in at least one location were discarded. For mRNA libraries, abundant cellular ribosomal RNAs were identified by mapping reads to a custom database, and removed before downstream analysis.

Identification of TE classes and differential expression of TE-derived piRNA and mRNA sequences

To include as many TE families as possible in our analysis, especially those that may be unique to the *D. simulans* genome, we examined both Repbase annotated TEs [2], and *de-novo* predicted TEs from the 12 *Drosophila* genomes generated by piler-DF [3],[4]. Unfortunately, considerable redundancy exists between Repbase curated *Drosophila* TEs [2], and the *de-novo* predicted TEs from piler-DF [4]. Additionally, the piler-DF database of consensus sequences is itself redundant, as these predictions were generated by looking for highly repeated sequences within genomes with no knowledge of true TE structure. To identify redundant sequences, all consensus TEs were compared to each other using blastn [5]. Those consensus sequences that were >90% identical across >90% of their length were categorized as being in the same class. Classes are identified as the Repbase-curated TE contained within this class (e.g. *roo*), or by the species that they were originally curated from (e.g. dsim.group.1) for those that did not contain a repbase sequence. A list of grouped repbase and piler-DF TE annotations can be found in Table S8.

The close sequence relationship between TEs of the same family makes it impossible to examine the activity of individual TE insertions. Differential abundance of TE-derived transcripts therefore was examined by pooling all reads that map to the same class. Reads that mapped to multiple TE classes were not considered as TE-derived reads. Raw read counts were used to examine differential abundance of TE classes between genotypes for both small RNAs and mRNAs using the R-packages edgeR [6] and DEGseq [7]. Non-TE derived reads were included in the analysis to account for differences in the total number of sequenced mRNAs or candidate piRNAs. TE-derived read counts for mRNA-seq and small RNA seq libraries from *D. melanogaster*, *D. simulans* and their interspecific hybrids are presented in Table S9.

For edgeR analyses of differential abundance of TE classes between genotypes, differences in sequencing depth and overall abundance of candidate piRNAs (small RNA libraries only) were normalized by equalizing library sizes using a quantile adjustment. Differential expression of each TE class was determined using an exact test similar to Fisher’s, but evaluated over a negative binomial distribution which is thought to be more appropriate to overdispersed data such those generated by RNAseq [8]. For DEGseq analysis of differential abundance of TE classes between genotypes, magnitude and statistical significance of differential abundance of TEs between treatments was inferred using an MA-based plot method with random sampling [7]. This method normalizes for differences in total read count when calculating the estimated difference in abundance between genotypes, but unlike edgeR, does not adjust raw read counts prior to analysis [7]. For both tests, p-values were corrected using the false discovery rate approach of Benjamini and Hochberg [9]. The two approaches yield qualitatively similar results for estimated fold differences in transcript abundance; however, for inference of differential abundance between treatments we present only the DEGseq analysis, as this generated a more normal distribution of estimated values. Quantile normalized values from edgeR were used for graphical comparisons of the abundance of sequences between libraries.

Both edgeR and DEGseq analyses effectively normalize to the size of the sequencing library (all mRNAs or only candidate piRNAs), which is a standard approach for mRNA-seq experiments and also has been used for analyses of piRNA populations from intraspecific hybrids [10]. This type of analysis reveals changes in the composition of the piRNA or mRNA pool by identifying differences in the relative abundance of individual TE families between genotypes. This differs from many piRNA analyses, which reveal absolute differences in TE-derived piRNA abundances between genotypes by normalizing to other populations of non-coding RNAs [11]–[13]. Such an approach was inappropriate to our data, as our ability to identify such sequences in *D. melanogaster* and *D. simulans* libraries would be biased by the difference in the quality of annotation between these two genomes (see Supplementary Discussion). Furthermore, normalizing to all candidate piRNAs better reveals differences in the relative abundance of piRNAs derived from individual piRNA clusters between hybrids and their parental pure species (Figure 5, Figure S4), because it accounts for the overall reduction in piRNA abundance in the interspecific hybrids.

piRNA cluster transcription

The piRNA clusters from *D. simulans* cannot be identified due to limited sequencing and assembly of its heterochromatin. Thus, all analyses of piRNA cluster transcription were performed by mapping piRNA reads to the 15 largest *D. melanogaster* piRNA clusters [14]. Mapping to each cluster was performed 3 different ways: 1) uniquely mapping reads only, 2) all mapable reads, divided by the number of other genomic regions to which they map, 3) all mapable reads. Mapping of each read was denoted by its 5’ end and read mapping was binned into 100 bp intervals. To account for differences in library size, mapped reads were normalized to the total number of candidate piRNAs in each sequencing library.

Calculation of the ping-pong signature

The ping-pong signature is the probability that a piRNA for a given TE class, randomly sampled from a library, will have an antisense binding partner sharing a 10bp overlap. This value was calculated by a custom perl script using the approach described in [10]. Briefly, all TE-derived piRNAs are first mapped to a consensus sequence. For each piRNA, the proportion of overlapping antisense binding partners whose 5’ end lies on the 10^th^ nucleotide is then determined. This value is subsequently summed across all piRNAs sampled for a given TE class, while incorporating the difference in sampling frequency between individual piRNAs. Finally, this sum is divided by the total number of piRNAs mapping to the TE class of interest.

mRNA-seq analysis of protein coding genes

To minimize the impact of differences between the assembly and annotation of the *D. melanogaster* and *D. simulans* genomes on our analysis of protein coding genes, we confined our analyses to those genomic regions represented in the UCSC full genome alignment, as in McManus *et al* [15]. These criteria excluded 5 known piRNA proteins from the data set: *argonaute-3*, *rhino*, *brother of Yb, sister of Yb,* and *squash*. These 5 sequences were aligned by hand between the *D. melanogaster* and *D. simulans* genomes in order to ensure the inclusion of equivalent exons. For all coding sequences, we considered only constitutively spliced exons to avoid potential differences in the identified splice variants between species [15].

mRNAseq reads were concurrently aligned to the exon databases described above of both *D. melanogaster* and *D. simulans*, and only those reads that mapped uniquely to a single coding sequence from either species were retained. We then compared transcript abundance in interspecific hybrids to both parental pure species using the MA-based plot method implemented in edgeR [6]. As with our TE analysis, *p*-values were corrected for multiple measures using the method of Benjamini and Hochberg [9]. Those coding sequences that exhibited a statistically significant increase in transcript abundance interspecific hybrids when compared to both parental pure species, with a magnitude of > 2-fold, were considered overexpressed. Reciprocally, those coding sequences that exhibited a statistically significant decrease in transcript abundance in interspecific hybrids when compared to both parental pure species, with a magnitude of > 2-fold, were considered underexpressed.

**SUPPLEMENTARY DISCUSSION A: Comparisons among intraspecific and interspecific hybrid dysgenesis models**

Two different intraspecific hybrid dysgenesis systems have been studied extensively in *D. melanogaster*: *I-element* and *P-element* dysgenesis [10],[16]–[21]. A third dysgenesis system, *Penelope*, has been thoroughly characterized in the distantly related species *D. virilis* [22]–[24]. Although our study focuses on the *I-element* dysgenesis of *D. melanogaster*, it is informative to draw comparisons between other intraspecific hybrid dysgenesis systems and the complex phenotypes that we observe in interspecific hybrids.

Like the *I-*element, both the *P-*element dysgenesis of *D. melanogaster* and the *Penelope* dysgenesis of *D. virilis* are characterized by a deficiency in the maternal cytotype of ovarian piRNAs derived from the dysgenic element [10],[20],[23],[24]. Furthermore, in all three intraspecific dysgenesis systems the inducer element is represented by transpositionally active elements in the paternal strain only [18],[19],[22]. Therefore, differences between strains in maternally deposited piRNAs and active TE families are hallmarks of intraspecific hybrid dysgenesis. This dramatically contrasts with interspecific hybrids, where TE derepression is not associated with interspecific differences in either ovarian piRNA pools or active TE families (Figure 1A,D).

Although TE transcript abundance has yet to be examined in *Penelope* dysgenesis, both the *I-* and *P-*element systems exhibit a few TE families (8 and 7, respectively) whose transcripts are more abundant in the dysgenic intraspecific hybrid ovaries when compared to the maternal strain (Figure 1B) [21]. As we note in the Discussion, in *I*-element dysgenesis no TEs are derepressed relative to both parental strains. Although a similar determination cannot be made for *P-*element dysgenesis because transcript abundance was not measured in the paternal strain [21], their ovaries cannot exhibit global transcriptional derepression of TEs if so few families are derepressed when compared to the maternal strain alone. Therefore, considering all the available data, patterns of TE expression in intraspecific dysgenesis appear fundamentally different from what we observe in interspecific hybrids, where transcript abundance of 32 TE families is exceptionally high when compared to both parental pure species (Figure 1A).

A major distinction between the *I-*element and *P-*element or *Penelope* dysgenesis systems is in their ovarian morphology. While *I-*element dysgenic ovaries resemble the interspecific hybrid used here in being wild-type in appearance, *P-*element and *Penelope* dysgenic ovaries are characterized by gonadal atrophy [17],[25]. Indeed, the nuage component Vasa, an important marker of all germline cells, is almost entirely absent from young *P-*element dysgenic ovaries, including cells present in the germline stem cell niche [21]. Because piRNA production occurs predominantly in the germline [11], the paucity or absence of these cells prohibits a meaningful analysis of the piRNA pool [10]. Thus, we cannot compare piRNA production during *P-element* or *Penelope* dysgenesis to piRNA production during *I-*element dysgenesis or in interspecific hybrids. Regardless, it is unlikely that ovarian atrophy results from disrupted piRNA silencing, because with the exception of *piwi*, piRNA pathway mutants have ovaries that are grossly normal in appearance (reviewed in [26]). Ovarian atrophy in *piwi* mutants, furthermore, is genetically separable from Piwi’s function in TE silencing [27].

**SUPPLEMENTARY DISCUSSION B: Differences in TE annotation and genome assembly between *D. melanogaster* and *D. simulans* and their potential impact on our study.**

A major challenge in the analysis of comparative genomics data is to control for differences in assembly and annotation between genomes. The *D. melanogaster* genome has high-quality 12x coverage of euchromatic regions [28], as well as a substantial amount of assembled heterochromatic regions [29]. *D. melanogaster* also has a long history of discovery and curation of TEs. The majority of repbase annotated TEs for *Drosophila* were discovered in *D. melanogaster* and use *D. melanogaster* for deriving their consensus sequence.

In contrast to *D. melanogaster*, the *D. simulans* euchromatic genome is a mosaic assembly of multiple strains, including *w^501^* sequenced at 2.9X coverage and 6 other strains sequenced at 1X coverage. Essentially no heterochromatic regions have been assembled for *D. simulans* [30]. While many TEs classes are shared between *D. melanogaster* and *D. simulans*, consensus sequences for most *D. simulans* TEs have not been reported. Additionally, the *D. simulans* genome has only recently been examined for extremely divergent or unique TE sequences [3],[4].

Mapping Reads to Transposable Elements

Because the genome assembly and TE annotation of *D. simulans* are far less complete than that of *D. melanogaster*, our primary concern is potential bias against identifying TE classes that are unique to *D. simulans*. *D. simulans*-specific TEs are especially important to identify because their inheritance in our study is strictly paternal. We explored two different methods to identify TE-derived mRNAs and small RNAs. First we mapped reads, allowing for up to 3 mismatches, to a database of consensus sequences for all annotated *Drosophila* TEs and repeats in repbase [2], as well as *de-novo* predicted TEs from piler-DF [4]. Second, we mapped reads, allowing for no mismatches, to a database of all annotated transposable element insertions in the genomes of both *D. melanogaster* and *D. simulans* [3].

The proportion of TE-derived sequencing reads identified using each approach is shown in Table S10. It is unclear for piRNAs which approach is superior at identifying TE-derived sequences. More *D. simulans* piRNAs are identified as TE-derived when mapping to all annotated insertions (71% versus 58%), whereas more *D. melanogaster* (69% versus 62%) and interspecific hybrid sequences (48% versus 46%) are identified as TE-derived when mapping to consensus sequences. By contrast, for all three libraries considerably more mRNAs are identified as TE-derived by mapping reads to all annotated TE insertions, rather than to consensus sequences (Table S10). This pattern is particularly striking for *D. simulans*, where there is a 3-fold increase in the number of TE derived reads identified by mapping to all annotated insertions, While it is difficult to know the cause of this discrepancy between approaches for maximized read mapping in mRNAs and piRNAs, mRNAs likely serve as more reliable indicators for the best mapping approach, as they are considerably longer than sequenced piRNAs (41 bp versus 23-31 bp) and therefore are less subject to spurious alignment as mismatches increase.

A second concern with the consensus mapping approach is that in interspecific hybrids, it is likely to be biased towards the identification of TEs and piRNAs inherited from the *D. melanogaster* mother, as these reads will have higher sequence similarity to most TE consensus sequences. To examine this, we identified TE-derived reads as specific to the *D. melanogaster* genome, specific to the *D. simulans* genome, or found in both genomes. For both mRNAs and piRNAs, the consensus-mapping approach identified more *D. melanogaster* specific TE-derived sequences in interspecific hybrids, while the insertion mapping approach identified a roughly equal proportion of TE-derived reads specific to the genome of each parental pure species (Figure S8). While we cannot be certain that the consensus mapping method is not detecting a real biological result, these observations are entirely consistent with the posited bias of mapping reads to a consensus sequence.

Based on our comparison of the consensus mapping and annotated insertion mapping approaches, we suggest that annotated insertion mapping suffers from less bias associated with the differences in quality and annotation of the two genomes. Therefore, comparisons of differential TE abundance in piRNAs and mRNAs presented in the main paper are from annotated insertion mapping. However, ~60% of TE classes observed as derepressed in interspecific hybrids are indentified by both approaches (Figure1A and Fig S9A-B), and the major conclusion of our study, that TE derepression in interspecific hybrids is not related to interspecific divergence in the piRNA pool, is supported by both mapping methods (*X^2^* = 1.38, *df* = 2, *p* = 0.50 Fig. S9C).

Antisense Fraction and Ping-pong Fraction

Calculating ping-pong and antisense fractions [10] requires mapping all piRNA reads to a consensus sequence to determine the strand of origin and 5’ end of the piRNA. For reasons discussed above, this almost certainly biases against the inclusion of divergent TE sequences from the *D. simulans* genome. Despite the apparent deficiency of *D. simulans* unique TEs, however, piRNAs sampled from *D. simulans* exhibit a more robust ping-pong fraction than those sampled from *D. melanogaster*, when considering the entire piRNA pool (Figure 6B-C), as well as when considering species-specific and shared sequences separately (Figure S10). If ping-pong amplification worked efficiently in hybrids, one would expect ping-pong fractions intermediate to those of each parental pure species. We instead find that ping-pong fractions to be severely reduced in hybrids, consistent with our conclusion that hybrids are defective for the ping-pong amplification cycle (Figure 6B-C, Figure S10).

piRNA cluster activity

We were unable to compare piRNA cluster activity between *D. melanogaster* and *D. simulans* because these clusters are either absent or only partially assembled in the *D. simulans* genome. To determine if this may have affected the major conclusion of our study, that interspecific differences in the piRNA pool do not explain TE derepression, we reanalyzed our data after excluding all reads uniquely mapping to *D. melanogaster* piRNA clusters. Of 272 TE classes represented within *D. melanogaster* piRNAs, only 5 elements changed significantly in abundance when uniquely mapping piRNAs were excluded from the piRNA pool (Figure S11A). Furthermore, when examining the relationship between interspecific differences in ovarian piRNAs and TE derepression in interspecific hybrids, these variables remain completely independent (*X^2^* = 0.18, *df* = 2, *p* = 0.91, Figure S11B)*.* We therefore conclude that reads uniquely assignable to piRNA clusters make a relatively small contribution to total piRNAs, and that the absence of *D. simulans* piRNA clusters does not create a significant bias in our inferences.

**References**

1. Langmead B, Trapnell C, Pop M, Salzberg SL (2009) Ultrafast and memory-efficient alignment of short DNA sequences to the human genome. Genome Biol 10: R25.

2. Jurka J, Kapitonov VV, Pavlicek A, Klonowski P, Kohany O, et al. (2005) Repbase Update, a database of eukaryotic repetitive elements. Cytogenet Genome Res 110: 462–467.

3. Clark AG, Eisen MB, Smith DR, Bergman CM, Oliver B, et al. (2007) Evolution of genes and genomes on the *Drosophila* phylogeny. Nature 450: 203–218.

4. Smith CD, Edgar RC, Yandell MD, Smith DR, Celniker SE, et al. (2007) Improved repeat identification and masking in *Dipterans*. Gene 389: 1–9.

5. Altschul SF, Gish W, Miller W, Myers EW, Lipman DJ (1990) Basic local alignment search tool. J Mol Biol 215: 403–410.

6. Robinson MD, McCarthy DJ, Smyth GK (2010) edgeR: a Bioconductor package for differential expression analysis of digital gene expression data. Bioinformatics 26: 139–140.

7. Wang L, Feng Z, Wang X, Wang X, Zhang X (2010) DEGseq: an R package for identifying differentially expressed genes from RNA-seq data. Bioinformatics 26: 136–138.

8. Robinson MD, Smyth GK (2008) Small-sample estimation of negative binomial dispersion, with applications to SAGE data. Biostatistics 9: 321–332.

9. Benjamini Y, Hochberg Y (1995) Controlling the false discovery rate - a practical and powerful approach to multiple testing. J R Stat Soc Series B Stat Methodol 57: 289–300.

10. Brennecke J, Malone CD, Aravin AA, Sachidanandam R, Stark A, et al. (2008) An epigenetic role for maternally inherited piRNAs in transposon silencing. Science 322: 1387–1392.

11. Malone CD, Brennecke J, Dus M, Stark A, McCombie WR, et al. (2009) Specialized piRNA pathways act in germline and somatic tissues of the *Drosophila* ovary. Cell 137: 522–535.

12. Klattenhoff C, Xi H, Li C, Lee S, Xu J, et al. (2009) The *Drosophila* HP1 homolog Rhino is required for transposon silencing and piRNA production by dual-strand clusters. Cell 138: 1137–1149.

13. Li C, Vagin VV, Lee S, Xu J, Ma S, et al. (2009) Collapse of germline piRNAs in the absence of Argonaute3 reveals somatic piRNAs in flies. Cell 137: 509–521.

14. Brennecke J, Aravin AA, Stark A, Dus M, Kellis M, et al. (2007) Discrete small RNA-generating loci as master regulators of transposon activity in *Drosophila*. Cell 128: 1089–1103.

15. McManus CJ, Coolon JD, Duff MO, Eipper-Mains J, Graveley BR, et al. (2010) Regulatory divergence in *Drosophila* revealed by mRNA-seq. Genome Res 20: 816–825.

16. Bucheton A (1979) Non-Mendelian female sterility in *Drosophila melanogaster*: influence of aging and thermic treatments. III. Cumulative effects induced by these factors. Genetics 93: 131–142.

17. Kidwell MG, Novy JB (1979) Hybrid Dysgenesis in *DROSOPHILA MELANOGASTER*: Sterility Resulting from Gonadal Dysgenesis in the *P*-*M* System. Genetics 92: 1127–1140.

18. Bingham PM, Kidwell MG, Rubin GM (1982) The molecular basis of *P*-*M* hybrid dysgenesis: the role of the *P* element, a *P*-strain-specific transposon family. Cell 29: 995–1004.

19. Bucheton A, Paro R, Sang HM, Pelisson A, Finnegan DJ (1984) The molecular basis of *I*-*R* hybrid dysgenesis in *Drosophila melanogaster*: identification, cloning, and properties of the *I* factor. Cell 38: 153–163.

20. Chambeyron S, Popkova A, Payen-Groschêne G, Brun C, Laouini D, et al. (2008) piRNA-mediated nuclear accumulation of retrotransposon transcripts in the *Drosophila* female germline. Proc Natl Acad Sci U S A 105: 14964–14969.

21. Khurana JS, Wang J, Xu J, Koppetsch BS, Thomson TC, et al. (2011) Adaptation to *P* element transposon invasion in *Drosophila melanogaster*. Cell 147: 1551–1563.

22. Evgen’ev MB, Zelentsova H, Shostak N, Kozitsina M, Barskyi V, et al. (1997) *Penelope*, a new family of transposable elements and its possible role in hybrid dysgenesis in *Drosophila virilis*. Proc Natl Acad Sci U S A 94: 196–201.

23. Blumenstiel JP, Hartl DL (2005) Evidence for maternally transmitted small interfering RNA in the repression of transposition in *Drosophila virilis.* Proc Natl Acad Sci U S A 102: 15965–15970.

24. Rozhkov NV, Aravin AA, Zelentsova ES, Schostak NG, Sachidanandam R, et al. (2010) Small RNA-based silencing strategies for transposons in the process of invading *Drosophila* species. RNA 16: 1634–1645.

25. Lozovskaya ER, Scheinker VS, Evgen’ev MB (1990) A hybrid dysgenesis syndrome in *Drosophila virilis*. Genetics 126: 619–623.

26. Klattenhoff C, Theurkauf W (2008) Biogenesis and germline functions of piRNAs. Development 135: 3–9.

27. Klenov MS, Sokolova OA, Yakushev EY, Stolyarenko AD, Mikhaleva EA, et al. (2011) Separation of stem cell maintenance and transposon silencing functions of Piwi protein. Proc Natl Acad Sci U S A 102 108: 18760–18765.

28. Celniker SE, Wheeler DA, Kronmiller B, Carlson JW, Halpern A, et al. (2002) Finishing a whole-genome shotgun: release 3 of the *Drosophila melanogaster* euchromatic genome sequence. Genome Biol 3: RESEARCH0079.

29. Hoskins RA, Carlson JW, Kennedy C, Acevedo D, Evans-Holm M, et al. (2007) Sequence finishing and mapping of *Drosophila melanogaster* heterochromatin. Science 316: 1625–1628.

30. Begun DJ, Holloway AK, Stevens K, Hillier LW, Poh Y-P, et al. (2007) Population genomics: whole-genome analysis of polymorphism and divergence in *Drosophila simulans*. PLoS Biol 5: e310.
